# Solid Lipid Nanoparticle-Based Calix[n]arenes and Calix-Resorcinarenes as Building Blocks: Synthesis, Formulation and Characterization

**DOI:** 10.3390/ijms141121899

**Published:** 2013-11-05

**Authors:** Imed Montasser, Patrick Shahgaldian, Florent Perret, Anthony W. Coleman

**Affiliations:** 1 INRAP, Technopôle de Sidi Thabet, Sidi Thabet 2020, Tunisia; E-Mail: imed.montasser@inrap.rnrt.tn; 2 School of Life Sciences, University of Applied Sciences Northwestern Switzerland, Muttenz 4132, Switzerland; E-Mail: patrick.shahgaldian@fhnw.ch; 3 Université Lyon 1, ICBMS UMR-CNRS 5246, CSAp, Villeurbanne F69622, France; E-Mail: florent.perret@univ-lyon1.fr; 4 LMI CNRS, UMR 5615, Villeurbanne 69622, France

**Keywords:** solid lipid nanoparticles, calix[n]arenes, calix-resorcinarenes, encapsulation, colloids, supramolecular, nanocarriers

## Abstract

Solid lipid nanoparticles (SLNs) have attracted increasing attention during recent years. This paper presents an overview about the use of calix[n]arenes and calix-resorcinarenes in the formulation of SLNs. Because of their specific inclusion capability both in the intraparticle spaces and in the host cavities as well as their capacity for functionalization, these colloidal nanostructures represent excellent tools for the encapsulation of different active pharmaceutical ingredients (APIs) in the area of drug targeting, cosmetic additives, contrast agents, *etc*. Various synthetic routes to the supramolecular structures will be given. These various routes lead to the formulation of the corresponding SLNs. Characterization, properties, toxicological considerations as well as numerous corresponding experimental studies and analytical methods will be also exposed and discussed.

## 1. Introduction

Solid lipid nanoparticles (SLNs) have gained significant attention as potential alternative colloidal particles. The use of the SLNs is an attractive innovation that is advantageous because the solid matrix of the lipid provides more flexibility in controlling the release process of the encapsulated ingredients [1–3]. The research groups of Müller in Germany and Gosgo in Italy were the first to investigate this type of colloidal system.

**Figure 1:**
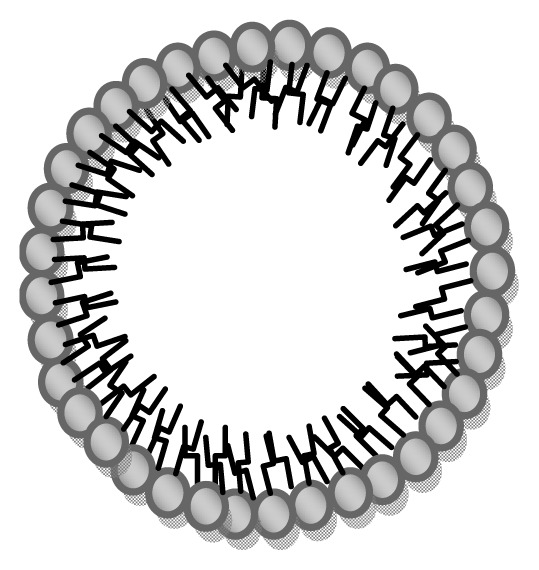
Proposed structure of solid lipid nanoparticles (SLNs), the interior structure varies from amorphous to crystalline.

SLNs are produced by replacing the liquid lipid (oil) of an oil/water emulsion by a solid lipid or blend of solid lipids, giving particle sizes in the range 80–1000 nm, and which are dispersed in water or aqueous surfactant solutions [4,5] (Figure 1).

SLNs are often produced by a melt emulsification followed by a hot or cold high-pressure homogenization (HPH) process. Briefly, the lipid phase is first melted at 5–10 °C above its melting point. The active ingredient to encapsulate is consequently dissolved or dispersed in the molten phase produced. Subsequently, a pre-emulsion is produced by stirring this melted phase in a hot surfactant solution. The pre-emulsion obtained is homogenized under high pressure to produce a hot nanoemulsion, which is further cooled down for the lipid recrystallization and the formation of SLN suspensions [6–8]. The cold HPH technique requires a first step of solid lipid melting in which the product to be encapsulated can be dissolved and/or admixed. Using liquid nitrogen or a dry ice, the lipid phase can be cooled down rapidly and solidified. By means of mortar milling, the solid lipid phase is ground down to nanoparticles and then dispersed in a cold aqueous surfactant solution to yield a pre-suspension that is homogenized at or below room temperature using the HPH [9–11].

SLNs can also be obtained also by precipitation of lipids using water-immiscible organic solvents [12,13], or semi-polar water-miscible solvents [14–16]. Solvent diffusion methods based on partially water miscible solvents were also investigated to prepare SLNs [17,18].

Different lipids can be used for the production of SLNs including triacylglycerols, acylglycerols, waxes and hard fats. Some examples of used lipids are given in the Table 1[19].

Lipophilicity, purity, and crystallinity of the lipid material, are important factors in the selection of lipid and the production process. The solubility of the product to be encapsulated in the lipid phase drastically affects the loading capacity [20–24].

In order to improve the solubility of some active ingredients, a small amount of liquid lipid (oil) can be mixed with the solid lipid phase to obtain nanostucturated lipid carriers (NLCs). However, if a large amount of oil is mixed, a different type of nanostructures can be produced. Here, the solubility of the oil in the solid lipid phase is exceeded leading to a phase separation and producing oily nanostructures within the solid lipid matrix [25,26].

The purity of the lipids used can affect the zeta potential and therefore the stability of the SLN suspensions. For Intravenous (IV) administration of SLN suspensions they should be stable and toxicologically accepted [27]. In addition, the purity of the solid lipids used plays an important role in the oral administration of the SLNs. In fact, the release of active ingredients from the SLNs in the gastrointestinal tract (GIT) depends on the lipase/co-lipase activity for the GIT digestion of the lipid matrix. The lipase/co-lipase complex leads to a degradation of lipids before absorption [19]. Lipid crystallinity is correlated with the encapsulation efficiency and the release rate.

In addition, these parameters are governed by the polymorphism of the lipid matrix. Polymorphism is the ability of the compound to crystallize in more than one distinct crystalline form with different internal lattices [28]. For instance, for SLNs produced with triacylglycerols (TAGs), the internal structure of the SLNs is related to the three-dimensional structure of the lipid matrix which can adopt the following structures upon crystallization: the hexagonal (α, H) phase, with a subcell lattice spacing of 0.415–0.42 nm, the orthorhombic perpendicular (β’, O_⊥)_ phase, with a subcell strong lattice spacing of 0.42–0.43 and 0.37–0.40 nm, or as the triclinic parallel (β, T_||_) phase with a subcell strong lattice spacing of 0.46 nm. Other polymorphic forms may also be found with complex acylglycerols including mixed acid TAGs or partial acylglycerols; for example, multiple β’ and β, sub-α or intermediate forms, usually mentioned as β_i_. The change in lipid structure can affect the expulsion during the storage and the release profile of the encapsulated product [29,30]. Moreover, the rate of polymorphic transformation depends on the length of the lipid chain and is the greatest for TAGs with short-chain fatty acids [31]. Once a stabilized SLNs formulation has been produced, the release is faster from α, than from β’ and β phases [28,32]. Differential scanning calorimetry (DSC) [33,34] and X-ray diffractometry (XRD) [34] are two widely used techniques to determine the crystallinity and polymorphic behavior of the components of the SLNs/NLCs. DSC provides information on the melting and crystallization behavior of all solid and liquid constituents of the particles, whereas XRD can identify specific crystalline compounds based on their crystal structure [35]. DSC utilizes the fact that different lipids possess different melting points and melting enthalpies. In XRD, the monochromatic X-ray beam is diffracted at angles determined by the spacing of the planes in the crystals and the type and arrangement of the atoms, which is recorded by a detector as a pattern. The intensity and position of the diffractions are unique to each type of crystalline material. XRD patterns can be used to predict the manner of arrangement of lipid molecules, phase behavior, and characterize and identify the structure of lipids and drug molecules [36,37]. However, best results are observed when SLNs/NLCs dispersions are investigated directly as solvent removal may change the modification.

While conventional chemical synthesis is capable of improving the production of the solid lipid nanoparticles based on lipid drug conjugates; using covalent linking [38], supramolecular chemistry combines the twin concepts of self-assembly and molecular recognition to generate novel nano-structured systems. Self-assembled amphiphilic supramolecular building blocks can spontaneously form nanoparticles, and they are currently being used as nanocarriers. Calixarenes, along with cyclodextrins and calixresorcinarenes, are the most commonly studied macrocycles for the production SLNs.

Generally, this type of supramolecular moiety looks like a flower vase or calyx krater and consists of several arene rings; hence, calix[n]arenes and calix-resorcinarenes, where “*n*” is the number of the arene rings present [39].

As cyclodextrins are natural supramolecular macrocyclic oligosaccharides and their use in the area of nanotechnology has been reviewed by many groups [40–42], this review will be devoted to the SLNs based on calix[n]arenes and calix-resorcinarenes derivatives initially investigated by the group of Coleman and later by the group of Shahgaldian. The specific capacity of such supramolecular systems to encapsulate different active ingredients both in the supramolecular cavity via molecular recognition and within the SLNs matrix could be an interesting alternative to the classical SLNs described above.

## 2. Solid Lipid Nanoparticles of Calix[n]arenes

### 2.1. SLNs of Para-Acylcalix[4]arenes

**Figure 2:**
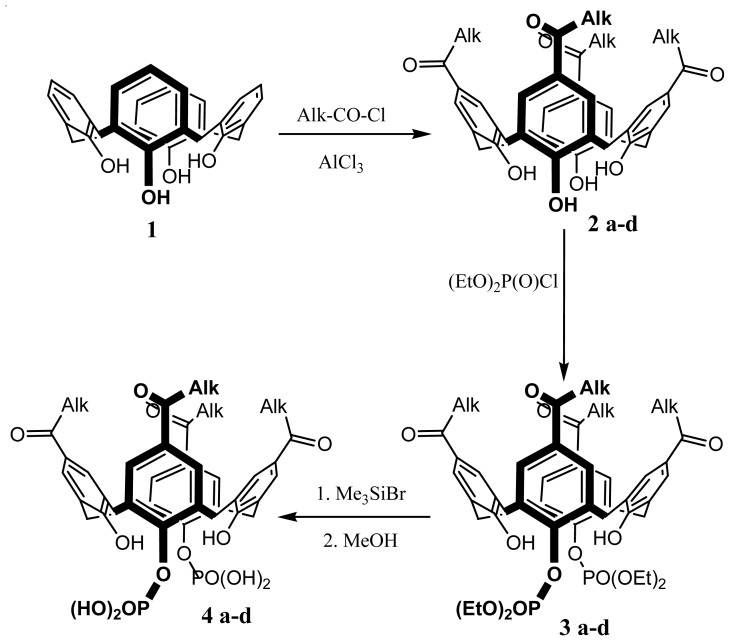
Synthetic route to different amphiphilic structures based on calix[4]arene. Alk = CH_3_(CH_2_)*_n_*, *n* = 4 (**a**), 6 (**b**), 8 (**c**), 10 (**d**).

The synthesis and the modification of various *para*-acylcalix[4]arenes has been reported by Shahgaldian *et al*. [43] (Figure 2). *Para*-acylcalix[4]arenes, **2a**–**d** were obtained using a total acylation at the *para* position of calix[4]arene, **1**, via a Friedel-Crafts reaction in presence of different acyl chlorides and aluminium trichloride as the Lewis acid. The regioselective 1,3-diphosphorylation of the *para*-acylcalix[4]arene, **3a**–**d** gave after deprotection the 1,3-di-phosphoric acids, **4a**–**d** in high yields.

SLNs based on **2a**–**d** were investigated by using the solvent displacement method [44,45]. Photon correlation spectroscopy (PCS), non-contact mode atomic force microscopy (AFM) showed that the diameter of the colloidal particles based on **2d** was 130–150 nm, and that the SLNs were highly stable, currently for over 10 years at 20 °C. For ^1^H NMR, the authors indicated that only broad signals for the alkyl chains were seen with a sharp peak at 0.85 ppm for the methyl head group, this observation was in agreement with the ^1^H NMR of solid lipid nanoparticles based on glyceryl behanate [46]. Therefore, it was concluded that the colloidal particles are solid with motion only at the chain termini.

In order to demonstrate the potential use of SLNs based on **2a**–**d** as topical delivery systems or cosmetic additives, their incorporation in different gel matrices such as carpobol 980, carpobol 2020, hyaluronic acid and xanthan was investigated using contact mode AFM imaging and PCS [47]. The results showed that the presence of the SLNs within the gels was easily detected in all cases. Moreover, these colloidal particles were dispersed in the sub-surface of gels as discrete particles of approx. 150 nm in diameter with no specific polymer-SLNs interaction.

In terms of formulation optimization and stability parameters, different factors have been investigated by the group of Coleman [48]. The studies showed that no change was observed in the particle sizes for different acyl chain lengths of **2a**–**d** (C_6_, C_8_, C_10_ and C_12_) when tetrahydrofuran (THF) was used as the organic phase. This result could be correlated with the crystal structures of **2a**–**d**[45]. Using different solvents as the organic phase such as acetone, THF, methanol and ethanol, the authors reported that no difference in term of particle sizes of the SLNs based on **2d** in the case of ethanol and THF. However, a larger particle size was obtained when methanol and acetone were used as the organic phase. This result was attributed to the differences in the chain arrangement when interacting with certain solvents. Such behavior was later confirmed by X-ray crystallography for the chain folding as a function of the solvent of crystallization [49].

The presence of certain surfactants for example, pluronic^®^F 68 led to a decrease in the particle size. This effect was related to the emulsifier and stabilizer roles of the surfactant, and was in a good agreement with results obtained for polymeric nanoparticles [50] and other SLNs based on commercial lipids [51]. In contrast to SLNs based on cyclodextrins [52], the SLNs based on **2a**–**d** were obtained without surfactant.

In view of the possible use of the SLNs based on **2a**–**d** for oral administration, their stability was tested at different pH values. It was shown that these colloidal systems are stable and the particle size remains invariant when the pH varies from 2 to 8. This high stability was in contrast to other SLNs, such as those based on compritol, where the surfactant/lipid ratio must be optimized to obtain suitable systems for gastro-intestinal administration [53].

Generally, an increase of the agitation speed during the emulsification step leads to a decrease in the particle size for polymeric dispersions [54]. However, for SLNs based on **2d** the size was independent of agitation speed a result which is advantageous for a scale up of this formulation process [54]. The influence of the viscosity of the aqueous phase was also investigated, with no significant variation of the particle sizes of the SLNs with increasing viscosity. In contrast, the viscosity of the aqueous phase plays an important role for the particle sizes of polymeric nanoparticles [50]. With regard to the concentration of the amphiphile in the final suspension, it was assumed that for low concentration of **2d** the equally sized droplets of the organic phase are dispersed into the aqueous phase, thereafter the solvent diffuses out into the water and the quantities of residual amphiphilic molecules then control the particle size. However, above the critical concentration (300 mg/L), the size remains invariant; this may be due to the particle’s mass being the determinant factor in the final size of colloids.

For SLNs based on **2a**–**d** to be used in the future as pharmaceutical or cosmetic preparations, certain post-formulation parameters should be respected [48]. The stability near the physiological ionic strength was tested, the results showed that the SLNs suspension based on **2d** remain stable and the particle size was invariant in solutions of sodium and potassium chloride, sodium phosphate, sodium carbonate and potassium nitrate. For other salts such as sodium carboxylate and potassium phosphate, the SLNs were less stable and an increase in the particle size of between 10% and 25% was observed. These results can be compared to those obtained for the SLN formulations based on compritol that were unstable in solutions of sodium, calcium and aluminum chlorides [53]. SLN formulations based on **2d** were also stable under UV, microwave irradiation, ultrasonic and thermal treatment. Therefore, these SLNs can be sterilized for IV administration.

**Figure 3:**
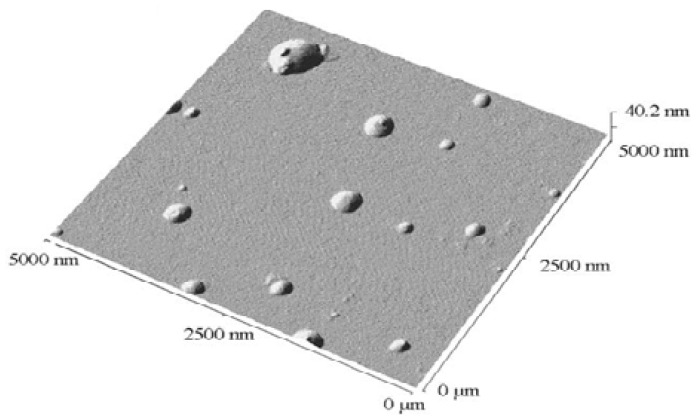
Non-contact mode atomic force microscopy (AFM) images of **2d**-based SLNs reconstituted after freeze-drying in a solution of glucose (2%) at 5 mm scan range.

Dry formulations represent a stable dosage form for oral administration in the form of Tablet and for an IV administration in the form of suspension or solution after re-dispersion in water. Thus such water soluble dispersible powders represent potential additives for cosmetic and topical preparations. For these reasons, freeze-drying of the SLN suspension based on **2a**–**d** was investigated. To avoid aggregation during the re-dispersion step [55], different cryprotectants such as glucose, fructose, mannose and maltose were investigated during the lyophilisation of the suspensions based on **2d**. No change in the redispersed particle size was observed using PCS for the different cryoprotectants even at high concentrations. The carbohydrates used formed a hydrophilic protective layer surrounding the SLN matrix. This layer may accelerate and improve the re-dispersion of the dried SLNs in water. Non-contact mode AFM of the re-dispersed SLNs after freeze-drying in an aqueous solution of glucose was investigated (Figure 3); the SLNs were present as individual spherical objects of 16 nm in height and 270 nm in diameter. This difference could be explained by the fact that the SLNs were partially embedded in a gel formed by glucose [56].

**Figure 4:**
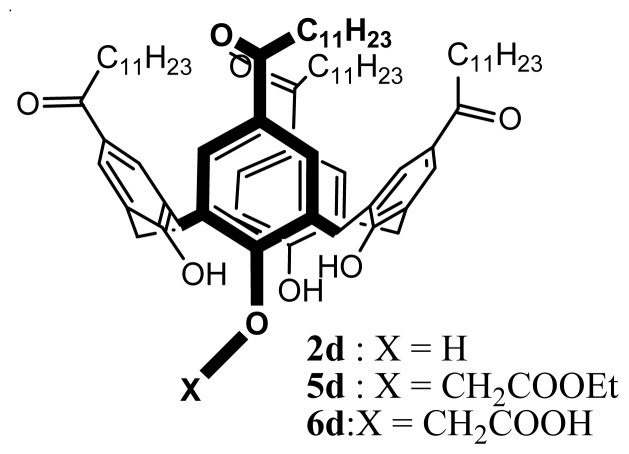
Formulae of **2d**, **5d** and **6d**.

Serum albumin and globulins represent the principal circulatory proteins with concentrations of about 46 and 27 g/L, respectively [57]. For a future use of the SLNs based on **2** as drug delivery systems, their interactions with bovine serum albumin (BSA) were investigated [57]. Three types of *para*-acylcalix[4]arene were tested **2d**, *para*-dodecanoyl-25-(ethoxy carbonyl methyloxy)-calix[4]arene (**5d**) and *para*-dodecanoyl-25-(2-carboxy-methyloxy)-calix[4]arene (**6d**) (Figure 4) [43,58].

**Figure 5:**
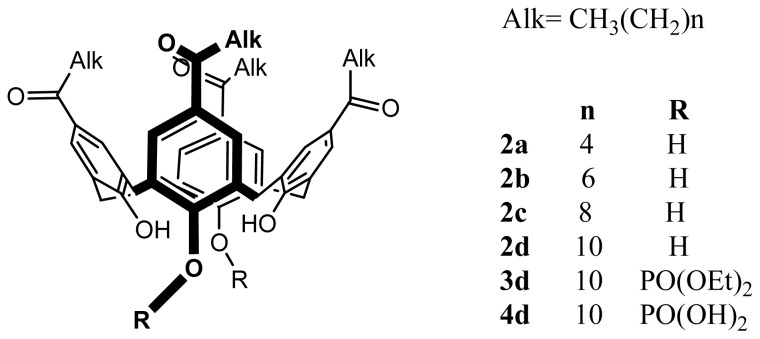
General formulae of the amphiphilic calix-arenes used in the haemolysis experiment.

From PCS analysis, the diameter of the SLNs in absence of BSA was 150, 183 and 193 nm for **2**, **5d** and **6d**, respectively. However, in the presence of BSA the diameter increases with increasing the BSA concentration in all the cases. For **2-**based SLNs the diameter increases from 152 to 166 nm with increasing concentration of BSA up to the physiological concentration of 40 mg/mL. This was attributed to the formation of a BSA capping layer at the surface of SLNs as observed for polymeric nanoparticles based on poly(*N*-isopropylacrylamide-co-methacrylic acid) [59]. The adsorbed layer thickness was estimated at 8 nm, equivalent to a double layer of BSA molecules. Regarding the **5d-**based SLNs, the diameter increased by 34 nm. This was attributed to a high affinity of BSA for the surface of **5d-**based SLNs; layer thickness is of the order of 17 nm. Adsorption could occur by two mechanisms; firstly, the BSA interacts with the SLNs with the same geometry as for **2** but with four layers adsorbed, or secondly, BSA is adsorbed in a single layer adsorption along its major axis (14 nm). For the **6d**-based SLNs, the size increase was 28 nm, here; the protein layer was estimated at 14 nm and is best seen as an adsorption along the major axis of BSA. This difference in the adsorption could arise from the variation of the hydrophilicity of the SLN surface and the specific interactions with the known amino acid binding sites of BSA (Arg117, Lys351 and Lys475) [57]. For more details regarding the interaction of the SLNs with BSA, non-contact mode AFM imaging was undertaken; no aggregation was observed for the SLNs in the presence of BSA which is a major advantage for the use these systems in IV drug delivery. The haemolytic effects of the SLNs based on derivatives of **2** have been investigated (Figure 5) [60].

The dispersions were prepared in phosphate buffer saline (PBS) at pH 7.4 and standard methods for the determination of the haemolytic effect were used. SLNs based on **2a**–**d** were stable in PBS and the particle size is given in Table 2. The haemolysis tests showed zero effects on erythrocytes (Table 3), undoubtedly due to a low affinity for these molecules and membrane lipids. This is in contrast to the result obtained with cyclodextrins [61–63] and represents an important advantage for the use of **2a**–**d** based SLNs as intravenous drug delivery systems.

**Figure 6:**
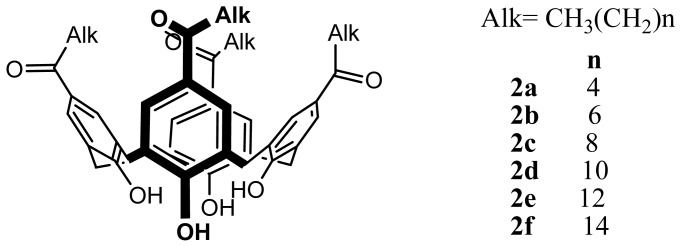
Chemical structures of amphiphilic calixarenes.

**Figure 7:**
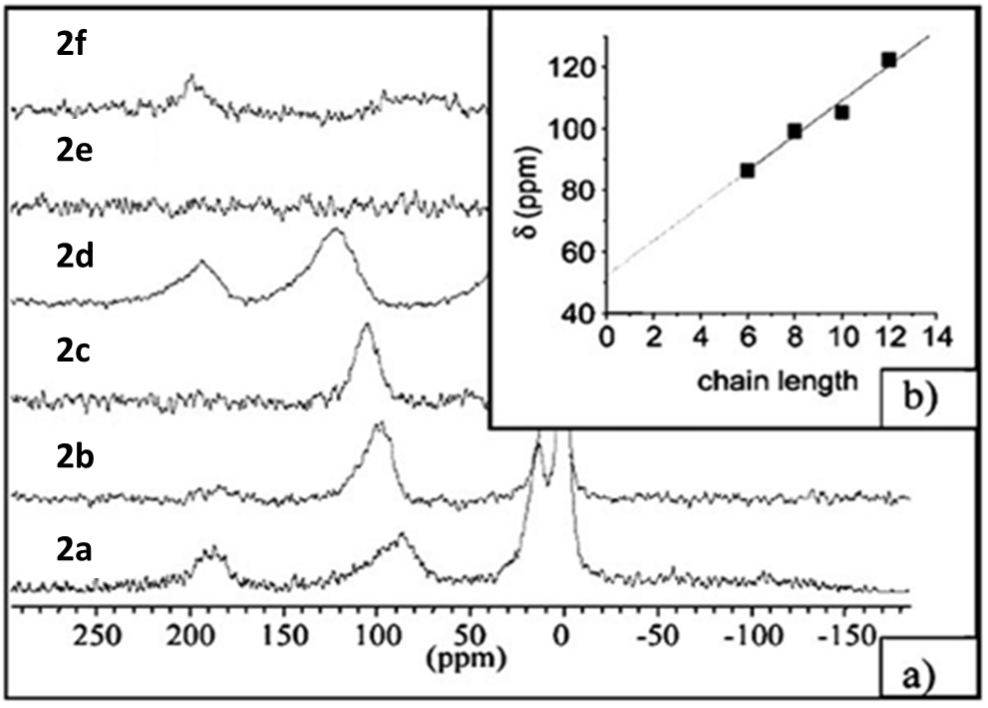
(**a**) Static ^129^Xe NMR spectra recorded under continuous flow of hyperpolarized xenon at an effective Xe pressure of 7 Torr and *T* 293 K; (**b**) Plot of the chemical shift of Xe in the host cavity *versus* the chain length of the amphiphilic calixarenes [68].

Because SLN suspensions have a low degree of crystallinity and thus the structure at the molecular level is largely unknown, NMR spectroscopy using hyperpolarized xenon (HP Xe NMR) [64–67] was investigated to obtain information regarding site distributions and therefore potential release profile as drug delivery systems [68]. Suspensions of SLNs were prepared as described above [44,48] and then freeze-dried [56] to obtain a powder suitable for analysis. The room-temperature NMR spectra of the SLNs prepared from **2a**–**d** (Figure 6) obtained using HP Xe produced in a continuous flow system are shown in Figure 7a.

The observed resonances at 0, ~20, ~80–130 and ~190 ppm were attributed to free Xe, Xe in the interparticle space, Xe interacting with the calixarene host cavity and Xe solubilized in the hydrophobic chains, respectively.

The increase of the shift of xenon interacting with the host cavity as a function of the number of carbons in the hydrophobic chains could be related to the reduction of void space for xenon in the host cavity. This relation between the shift and the chain length of the different *para-*acylcalix[4]arenes is represented in the Figure 7b. An extrapolation to zero leads to a chemical shifts about 52 ppm, which is very close to the previously value obtained in the case of short chain corresponding to *para*-tert-butylcalix [4]arene (~60 ppm) [69].

**Figure 8:**
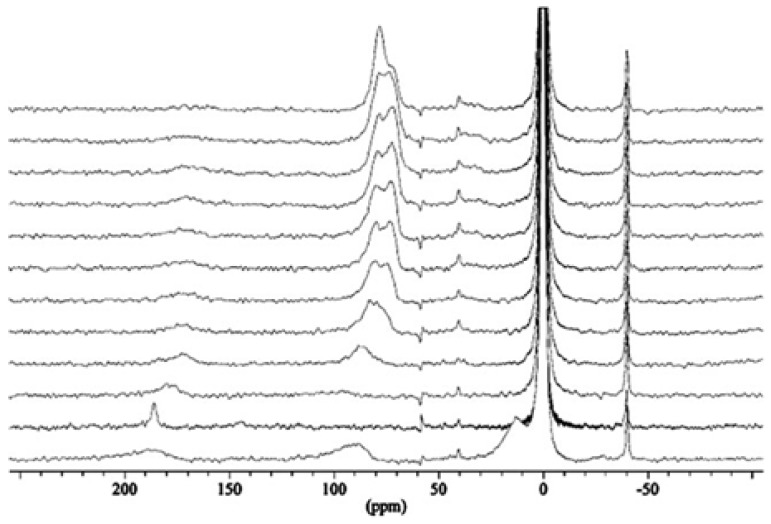
*In situ* transformation of **2a** SLNs (**bottom**) after a pulse of methylene chloride (next to **bottom**) as monitored by continuous flow hyperpolarized ^129^Xe MAS NMR. Consecutive spectra were recorded every 4.5 min at room temperature. Sharp signals at ±40 ppm are spinning sidebands [68].

To study the interaction of the SLNs with small guest molecules, methylene chloride was used as an additive to the ^129^Xe/N_2_/H_2_ gas mixture; the results are shown in Figure 8. When methylene chloride and the Xe were adsorbed together, a small sharp signal at ~190 ppm arises from Xe dissolved in the hydrophobic chains. After the methylene chloride pulse the spectra indicates clearly the return of Xe to the host cavity, and the single asymmetric signal (~80 ppm) was changed into two closely spaced and more shielded lines (*δ*_iso_ = 78.6 and *δ*_iso_ = 73.6 ppm), indicating that the Xe is localized in two inequivalent sites.

**Figure 9:**
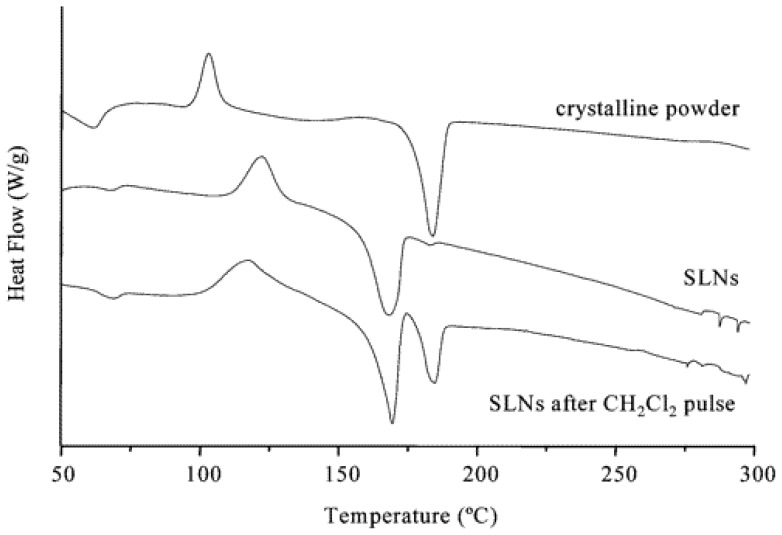
Differential scanning calorimetry traces for *para*-hexanoyl calix-[4]arene **2a**[68].

DSC studies of the crystalline powder were obtained before and after the CH_2_Cl_2_ pulse (Figure 9). The results show the presence of two distinct phases. The treatment of the SLNs with CH_2_Cl_2_ led to a transition from a state where the hydrophobic chains are included into the adjacent calixarene cavity to another state where the hydrophobic chains are expelled from the adjacent host cavity. Thus, the resulting material was composed of a partially ordered shell with increased void space.

These results illustrate three key features; firstly, host cavities were present at the surface of the SLNs and accessible to small chemical specii such as Xenon and CH_2_Cl_2_. The molecules were located in three areas: adsorbed on the surface, included into the cavities and entrapped in the SLN matrix, with each location giving a different release profiles. Secondly, the host cavities are loaded with hydrophobic chains of adjacent calixarenes, thus blocking the inclusion of the guest molecules. Thirdly, the pulse of a guest molecule led to the reorganization of the structure and therefore an increase the void site number for sorption.

**Figure 10:**
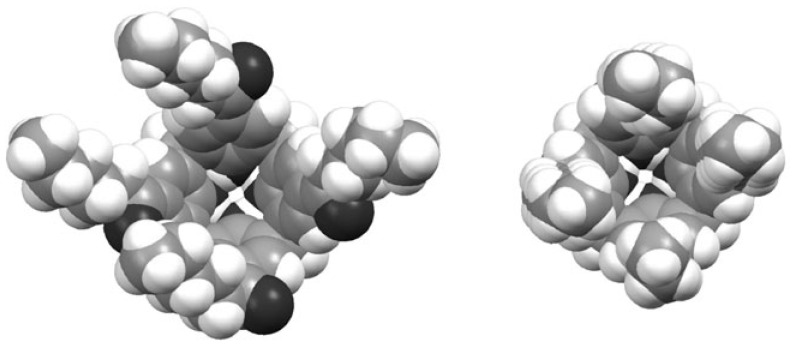
Top view to the cavity of *para*-hexanoylcalix[4]arene **2a** (**left**) and *para*-hexylcalix[4]arene (**right**) [70].

Encapsulation in the solid state of different guests within the host cavities of the *para*-acylcalix[4]arenes has been previously reviewed by Coleman *et al*. [70]. The molecule preferentially assumes a cone conformation in the solid-state, with a cavity potentially available for inclusion. The carbonyl group of the acyl chain serves as an extension of the host cavity of the calix[4]arene. The four carbonyl (C=O) groups fix the positions of the two first carbon atoms of each chain in the plane of the adjacent phenyl rings and thus control the cavity accessibility. Reduction of the carbonyl groups to methylene functions closes the cavity and prevents inclusion of guests as seen for *para*-hexylcalix[4]arene (Figure 10) [70].

Depending on the size and on the polarity of the guest as well as the crystallization medium, three types of solid structures are formed: (i) an interdigitated structure or partially self-included complex; (ii) open-container or head to tail complex; (iii) nanocapsular or tail-to-tail structure.

**Figure 11:**
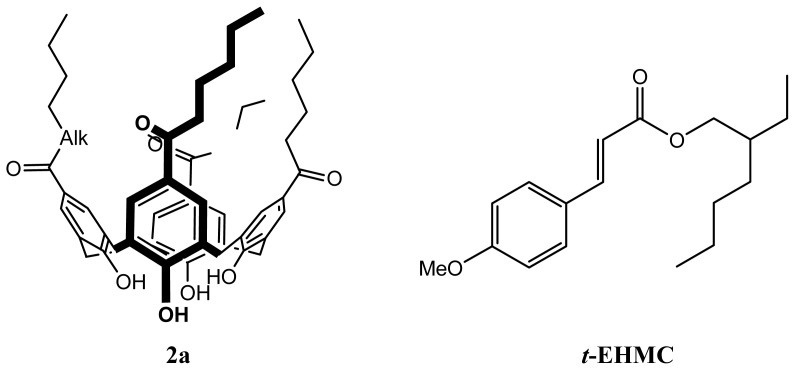
Structures of *para*-Hexanoyl Calix[4]arene (**2a**) and *t*-EHMC.

Aiming for a cosmetic application of the SLNs based on **2a**, encapsulation of a sunscreen blocker (*trans*-2-ethylhexyl-4-methoxy-cinnamate (*t*-EHMC)) was investigated (Figure 11) [71].

**Figure 12:**
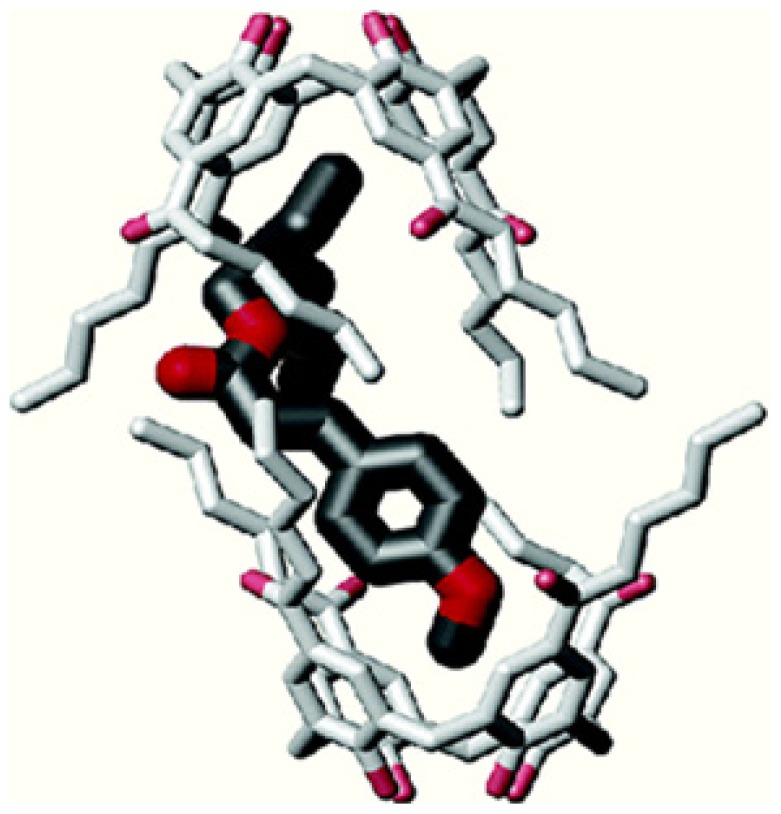
Capsular structure of inclusion complex of *para*-hexanoyl calix[4]-arene with *t*-EHMC (2*2a. *t*-EHMC). Disorder and H-atoms are omitted for clarity [71].

From solid state studies [72–74], the authors showed that the complex contains two molecules of p*ara*-hexanoylcalix[4]arene enclosing one molecule of *t*-EHMC inside a nanocapsular structure (Figure 12).

**Figure 13:**
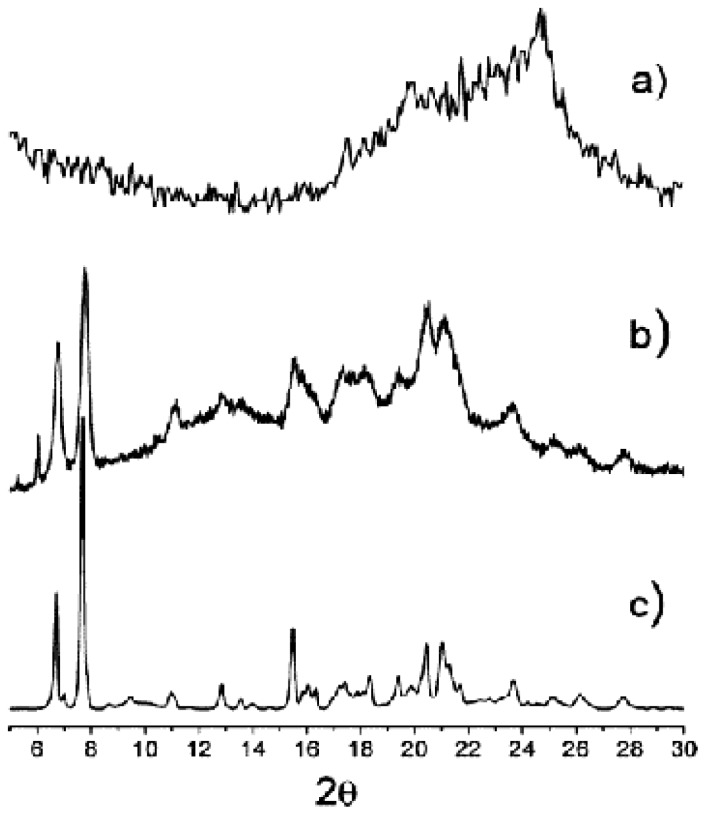
Powder XRD patterns of **2a** SLNs: unloaded SLN (**a**); SLNEHMC-1.2 (**b**); and crystalline inclusion complex 2*2a.1*t*-EHMC (**c**) [71].

Comparison of powder X-ray diffraction (XRD) patterns of unloaded SLNs, SLNs loaded with *t*-EHMC, and the crystalline complex 2.**2a**.1*t*-EHMC (Figure 13) showed that the SLNs loaded with *t*-EHMC are crystalline materials with a structure close to that of the complex 2.**2a**.1*t*-EHMC.

**Figure 14:**
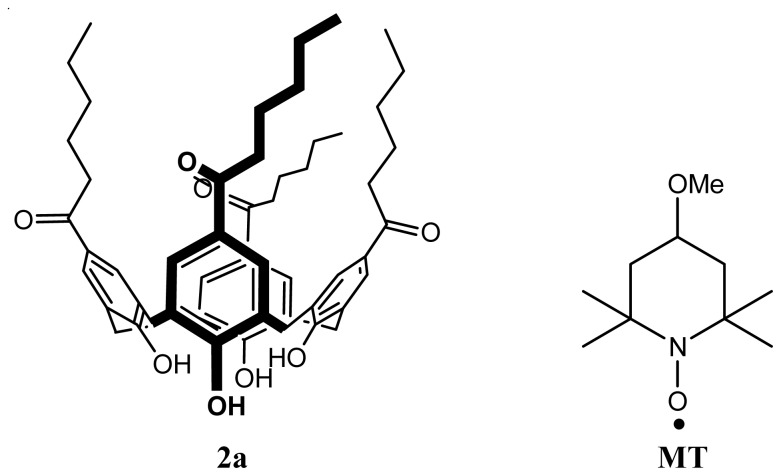
Structures of *para*-hexanoyl calix[4]arene (**2a**) and MT.

With regard to possible use of the systems in magnetic resonance imaging (MRI), the encapsulation of the stable paramagnetic radical (4-methoxy-2,2,6,6-tetramethylpiperidine-*N*-oxyl “MT”) has been investigated (Figure 14) [75].

**Figure 15:**
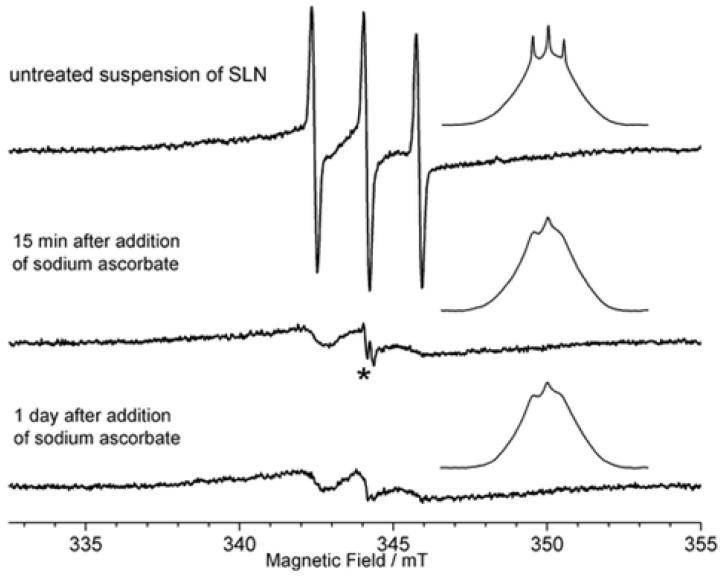
Electron spin resonance (ESR) spectra of SLN of **2a** loaded with MT upon treatment by the solution of sodium ascorbate at room temperature in buffer solution of pH 7.4. Insets: integrated spectra. The asterisk denotes the ascorbate anion-radical [75].

SLNs loaded with MT were prepared using the solvent displacement method [44,48] with a ratio for calixarene and MT of 1:1. The obtained suspension was then lyophilized for further studies. The multi frequency electron spin resonance (ESR) study was also investigated. In this case, the freeze-dried SLNs were re-dispersed in water using applying ultrasound and then filtrated for analyzis. The X-band ESR reveals that MT is present in two forms: as a free radical represented by sharp lines and as an encapsulated radical in the SLNs represented by broad lines (upper trace in Figure 15). The poor signal-to-noise ratio in the spectra was attributed to the efficiency of the re-dispersing step of the SLNs in water, the absence of cryoprotectants during the freeze-drying process prevents a good re-dispersion of the SLNs [56]. The addition of a reducing agent to the MT loaded SLN suspension removes the non-encapsulated radical and only the encapsulated MT is detected, with stability over days, indicating that the encapsulated nitroxide in not accessible to the reducing agent.

In conclusion, the encapsulation of free radicals into the SLNs studied here can be considered as a first step towards the design of more sophisticated structures, where the properties of host and guest can be adjusted for each particular application. As examples, the authors envisage new magnetic materials, MRI contrast agents, in situ pH probes, or models for oxidation stress studies.

### 2.2. Attempts to Produce SLNs of *Para*-Acylcalix[6]arenes and *Para*-Acylcalix[8]arenes

After the *para*-acylcalix[4]arenes the group of Coleman attempted, without success, to use *para*-acylcalix[8]arenes [76] and *para*-acylcalix[6]arenes [77] to formulate SLNs.

However, in contrast to **2a**–**d**[45] there are very considerable differences in the behaviour of these compounds at the air-water interface both regarding the molecular areas and the collapse pressure, suggesting a quite different form of self-assembly.

A second functionalization at the lower rim was also investigated using different anionic and nonionic groups [78] such as carboxymethoxy (anionic), carboxypropoxy (anionic), 4-sulfonatobutoxy (anionic), ethoxycarboxymethoxy (nonionic), ethoxycarboxypropoxy (nonionic), 2-methoxyethoxy (nonionic) and 2-(2-methoxy)diethoxy (nonionic). The results reveal that certain compounds having a *para*-octanoyl substituent form stable monolayers and have strong tendency to self-organise into 3D structures at the air-water interface. However, whilst standard solvent diffusion methods were ineffective, these molecules self-disperse in water to yield dispersions of 260 nm diameter. The interactions between *O*-4-sulfonatobutoxy-*para*-octanoylcalix[8]arene and a series of serum albumins have been studied by dynamic light scattering, and specific adsorption of this calix[8]arene derivative onto the proteins was observed [78].

Moreover, The anionic derivatives *O*-4-sulfonatobutoxy-*para*-ocatanoylcalix[8]arene as well as the *O*-carboxymethoxy-*para*-ocatanoylcalix[8]arene have been shown to possess anticoagulant properties and no haemolytic toxicity [78]. Regarding the *para*-acylcalix[6]arenes, molecules bearing butanoyl, hexanoyl and octanoyl chains have been synthesized by Friedel-Crafts acylation of the parent calix[6]arenes [77]. Per-substitution at the phenolic faces was achieved to yield the methoxy-diethoxy, ethoxycarbonylmethoxy, methoxycarboxylic acid and butoxysulphonate derivatives. For the para-acylcalix[6]arenes no stable monolayers can be formed at the air–water interface. However, stable monolayers are formed with the methoxy-diethoxy, ethoxycarbonylmethoxy, methoxycarboxylato compounds [77]. Finally, all these compounds showed no solubility in organic solvents to use for the solvent displacement process to formulate SLN_(s)_[79].

### 2.3. SLNs of *Para*-Acylcalix[9]arenes

**Figure 16:**
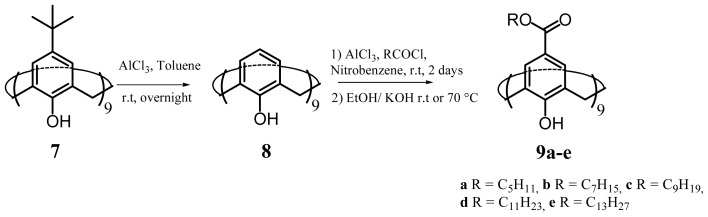
Synthetic route to the amphiphilic *para*-acyl-calix[9]arenes, **9a**–**e**.

The synthesis of **9a**–**e** (Figure 16) has been achieved using the total substitution of *para*-*tert*-butylcalix[9]arenes (**7**) [80]. It was noted that the complete removal of *tert*-butyl-groups in presence of aluminium trichloride as a Lewis acid using a modified standard method [81] to yield calix[9]arenes was the key step. The total para-acylation step was carried out under Friedel-Crafts conditions and the desired products were obtained after saponification of phenolic ester groups occurred in some cases [76].

The five obtained products (**9a**–**e**) showed good solubility in organic solvents such as THF and ethanol and were capable of forming SLNs by the solvent displacement method [44,48].

Characterization of these colloidal particles was carried out using transmission electron microscopy (TEM) and PCS [79]. The result shows particles as aggregated and slightly flattened circular objects with an average diameter of approximately 200 nm in the case of SLNs obtained from **9a** and 80 nm when obtained from **9e**.

It was shown that the size decreases when the length acyl chain increases (from C_4_ to C_12_) [79]. This result was in contrast to that obtained with the SLNs based on **2a**–**d**[48]. Regarding the influence of the organic phase, it was observed that in the case **9a** a stable colloidal suspension was obtained with THF and acetone, and in the case of acetone smaller objects are observed, however, with ethanol the colloidal suspension was unstable after 7 days. This result was attributed to the difference in the chain arrangement in presence of certain solvents and it is in complete contrast to the result obtained in the case of **2d**, where the SLNs obtained using acetone showed a larger size [48].

With the surfactant (Pluronic ^®^F68) in the case of **9e**, a small size increase was observed up to 10%, above this value a large increase was observed indicating flocculation. This observation was in contrast with the result found in the case of **2d**, where the surfactant played an emulsifier role to decrease the size [48].

As observed in the case of **2d**, no change in the particle size was also found in the case of **9a** when the speed of agitation is increased from 250 to 1250 rpm. Again this result is a perfect agreement with low cost and easy scale up of this formulation process [54].

The influence of the final concentration of **9a**, as well as the viscosity of the aqueous phase was studied. It was observed that the particle size remains invariant when the viscosity of the aqueous increases from 1.01 to 2.20 cP. However, an increase in the size was observed when the final concentration of the amphiphilic increases from 100 to 500 mg/L in the organic phase. The previous work on **2d** showed the same result regarding the effect of the viscosity.

Numerous post formulation parameters were studied to demonstrate the possible use of this kind of colloidal particles for future biomedical exploitations [79].

Firstly, the influence of various mono and divalent ions on the stability of the SLNs based on **9a** was measured in presence of NaCl, KCl, MgCl_2_ and CaCl_2_. At time 0, no change in the particle size was observed except for KCl at 50 mM for which an increase in the size of 25% was seen. After 24 h, Mg^2+^ at 1 mM and Ca^2+^ at 2 mM caused an increase in the particle sizes and flocculation of the suspension occurred above these concentrations. Regarding the pH stability, it was observed that the SLNs based on **9a**,**e** remain stable in the pH range from 3 to 12.

As seen for the SLNs based on **2d**, SLNs based on **9a** were also stable under UV, ultrasonic irradiation and thermal treatment. These results should allow sterilization of such nano-particles without degradation.

As indicated previously freeze-drying is the most commonly method to remove water from a colloidal suspension [56] increasing preservation efficiency [82]. Addition of cryprotectants to the SLNs suspension is often used before the freeze-drying process in order to conserve size and activity [83,84]. Regarding the freezing-unfreezing cycles, the SLNs suspension based on **9a** was stored at −15°C during 12 h and then unfrozen at room temperature. The re-dispersed SLNs were unstable even after one cycle. For this reason, freeze-dying in presence of different carbohydrate cryoprotectants [79] was investigated. In term of particle size, it was observed that the size did not change when the concentration of cryprotectant was equal to or greater than 2% (*w/w*). As with **2a**–**d** the carbohydrates can form a capping layer around the SLNs, such a hydrophilic layer may also accelerate the re-dispersion step in water [48].

The interaction of the SLNs based on **9a** with human serum albumin (HSA) was investigated to determine the initial possibility to use this kind of colloidal particles in the biomedical area. The SLNs suspension was diluted in aqueous solution of HSA in order to obtain a final concentration of HSA ranging from 1 to 40 g/L and analyzed using PCS [79]. The results revealed that the particle sizes increases when the concentration of HSA increases from 1 to 10 g/L, above this value, the particle size was invariant. HSA can form a capping layer as already observed in the case of polymeric nanoparticles [59]. The protein layer was estimated at 14 nm in depth, with either a single layer formed along the long axis or less probably by formation of a layer of four HSA molecules.

The encapsulation capacity has been tested using **9a** and acridine as fluorescent probe [79]. It was shown that the emission wavelength was changed from 425 nm in water to 475 nm after encapsulation. This change in emission was accompanied by an increase in the fluorescence intensity showing a clear effect of the local environment about the fluorophore as a result of encapsulation; the spectra remain stable during 24 h.

### 2.4. SLNs of Other Amphiphilic Calix[4]arene Derivatives

#### 2.4.1. SLNs of Phosphorylated-Calix[4]arenes, **14**–**15**

**Figure 17:**
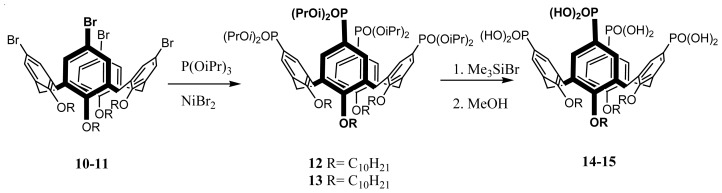
Synthetic route to the compounds **14** and **15**.

The amphiphilic dihydroxyphosphonyl-calix[4]arenes, **14**–**15**, were prepared from the previously synthesized tetrabromotetraalkyl-calix[4]arenes [85] (**10**–**11**) according to the literature procedure [86] via an Arbuzov reaction yielding the intermediate di-isopropyl phosphites (**12**–**13**), followed by de-protection (Figure 17).

SLN suspensions based on amphiphilic dihydroxyphosphonyl-calix[4]arenes (**14**–**15**) were prepared using the solvent displacement method described above [44,48]. The obtained colloidal suspensions were characterized by using PCS and AFM [86].

**Figure 18:**
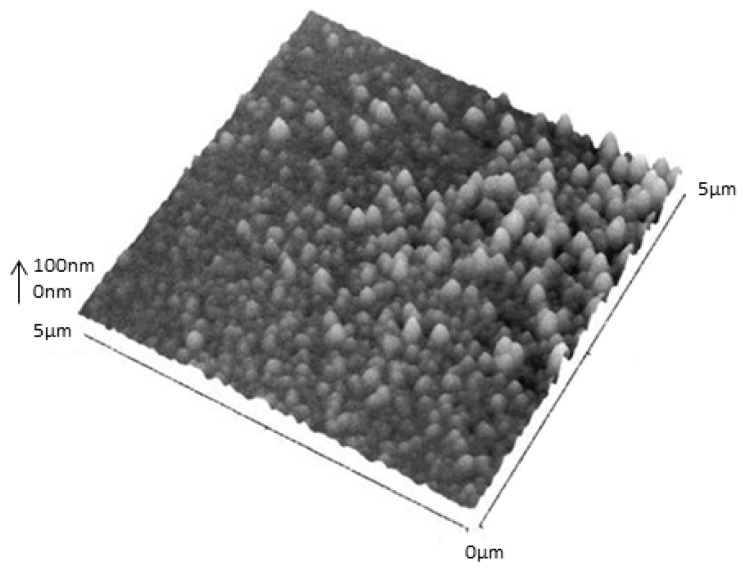
Noncontact mode AFM image of the solid lipid nanoparticles formed by **14** on mica at 5 × 5 μm scan size, *z* axis is 94 nm. A 20 μL portion of a suspension of the SLNs was deposited, and imaging was performed after drying for 24 h at 25 °C [86].

The diameter of the SLNs in pure water was 170 nm for **14** and 137 nm for **15**. Using noncontact mode AFM imaging, for **14** (Figure 18), the particles appeared as individual monodisperse flattened spheres with diameter of about 210 nm and height of about 60 nm.

**Figure 19:**
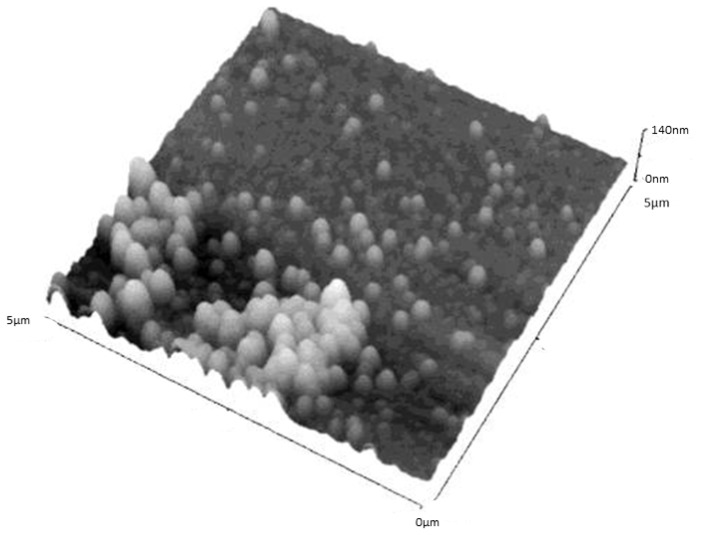
Non-contact mode AFM image of the solid lipid nanoparticles formed by **15** on mica at 5 × 5 μm scan size, *z* axis is 140 nm. A 20 μL portion of a suspension of the SLNs was deposited, and imaging was performed after drying for 24 h at 25 °C [86].

For SLNs based on **15** (Figure 19), aggregation occurred at the center of the dried droplet; however individual separated particles were present around the perimeter. The particles were again flattened spheres with diameter of about 190 nm and height of about 45 nm. The height difference between **14** and **15** was ascribed to the fact that the structure based on shorter alkyl chain (**14**) may have been slightly more rigid, with less flattening of the structures. In all the cases the observed heights are much greater than those observed for other vesicular nanostructures based on cyclodextrins [87] and calix-resocinarenes [88]. This observation confirms the solid structure of this kind of colloidal particles.

**Figure 20:**
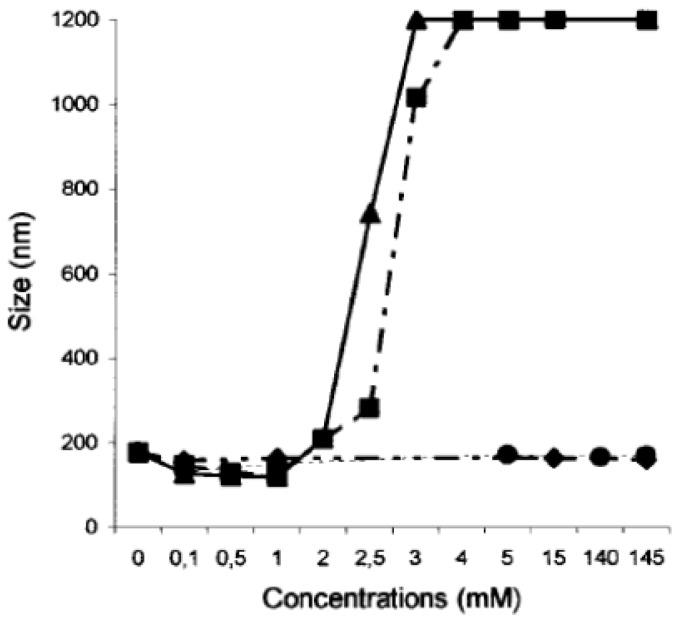
Variation of the diameter of solid lipid nanoparticles formed by **14** in the presence of varying concentrations of Na^+^ (♦); K^+^ (•); Mg^2+^ (▪); and Ca^2+^ (▴) [86].

**Figure 21:**
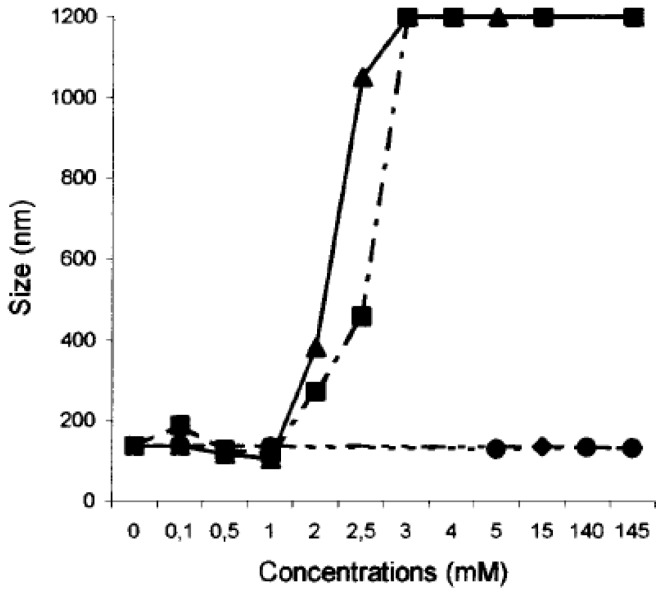
Variation of the diameter of solid lipid nanoparticles formed by **15** in the presence of varying concentrations of Na^+^ (♦); K^+^ (•); Mg^2+^ (▪); and Ca^2+^ (▴) [86].

With a view to future biomedical applications, the interactions of the SLNs with the physiological cations, Na^+^, K^+^, Mg^2+^ and Ca^2+^ has been evaluated at concentrations of extracellular and intracellular levels [86] (Figures 20 and 21). The results showed no change in term of size when the SLNs based on **14** and **15** were mixed with K^+^ and Na^+^ at concentrations up to physiological levels. For Mg^2+^ and Ca^2+^ a slight but significant decrease in the size was observed up to physiological levels; at higher concentrations aggregation was observed.

**Figure 22:**
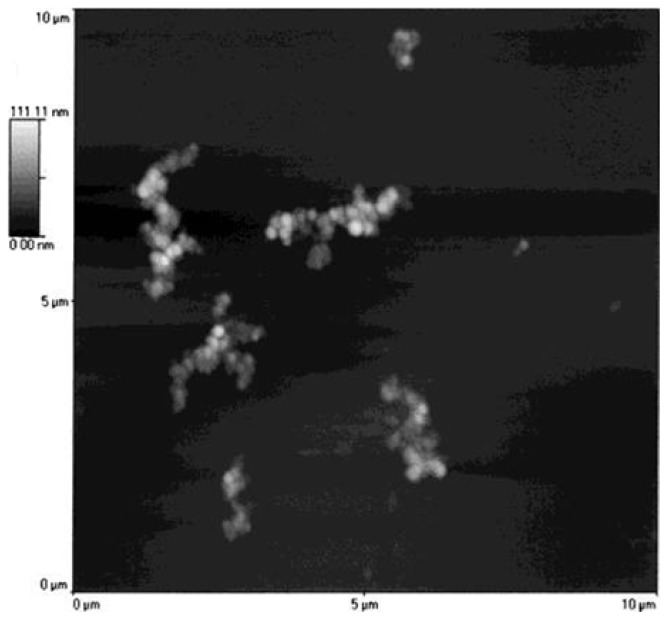
Noncontact mode AFM image of the solid lipid nanoparticles of **15** in the presence of 3 mM CaCl_2_ on mica at10 μm scan size [86].

Non-contact mode AFM imaging showed clearly aggregation when compared the SLNs based on **15** alone (Figure 19) and in the presence of 3 mM of CaCl_2_ (Figure 22). It can be seen that the aggregates are about 2 μm in length and 0.5 μm in width, and this result is in perfect agreement with the aggregation (>1 μm) observed in the PCS analysis. A similar study was undertaken using SLNs based on acylated β-cyclodextrin derivatives, and it was observed that the aggregation phenomenon occurred in presence of all cations even at concentrations as low as 0.1 mM [89].

#### 2.4.2. SLNs of *Para*-Amino-Substituted-Calix[4]arenes, **17**

**Figure 23:**
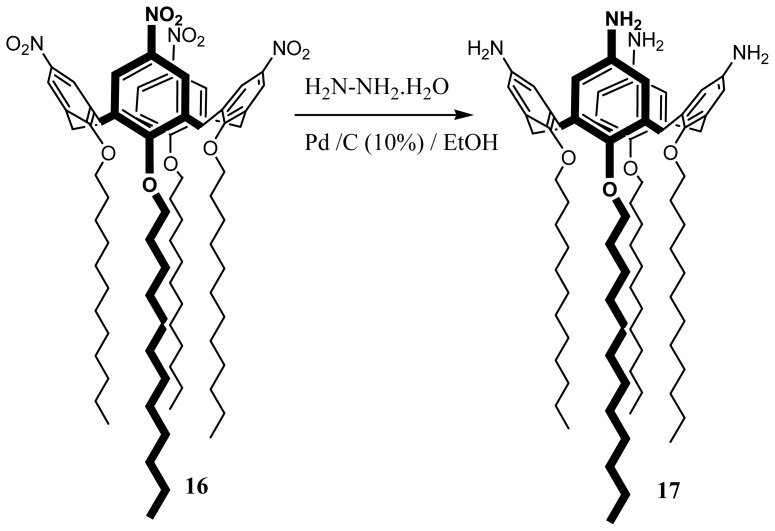
Synthetic route to **17**.

Amphiphilic 5,11,17,23-tetramino-25,26,17,28-tetradodecyloxycalix[4]arene (**17**) has been synthesized from the *para*-tetra-nitro derivative previously prepared by *ipso* nitration [90] and a catalytic reduction reaction [91,92] (Figure 23).

**Figure 24:**
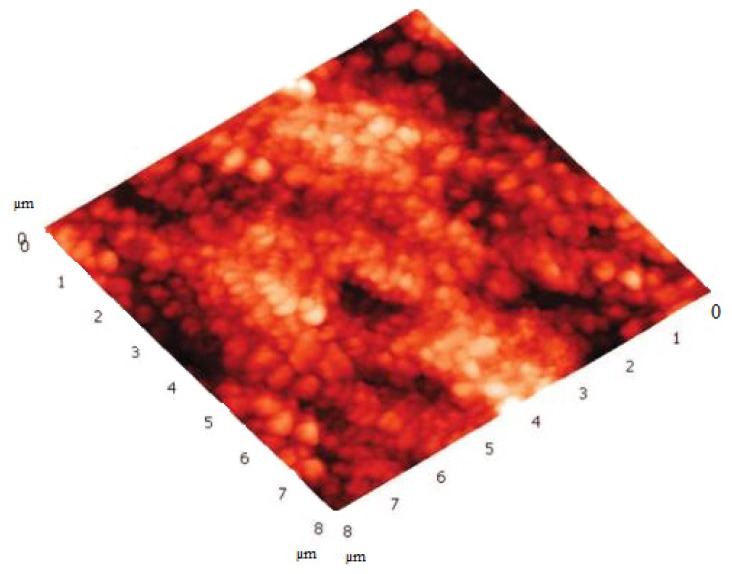
Atomic force micrsocope image of **17**-based SLNs spread on mica and imaged in air in noncontact mode [92].

SLNs based on **17** were prepared according to the solvent displacement method by using THF as the organic phase in the absence of surfactant [44,48,92]. In term of particle sizes, PCS showed that the suspension was formed of particles 190 nm in diameter. The particles present a net charge of +13.2 mV, a value which confirms the possible repulsive interactions between the positively charged ammonium groups at the surface of the particles. For a confirmation of the solid structure of these particles, non-contact mode AFM studies were carried out. The results showed (Figure 24) that the particles are present as round shaped objects with a size of 80 ± 10 nm in height and 200 ± 20 in width [92].

Due to their cationic nature, the SLNs based on **17** were capable of complexing DNA [91–96]. Aiming to open new prospects to use these SLNs as gene delivery systems [97], cell transfection using a layer by layer (LbL) coating technique was investigated, and the SLNs were loaded at their surface with DNA and chitosan [98]. Chitosan is a biopolymer capable of adhering to negatively charged surfaces such as skin and mucosa and possessing wound healing proprieties [99]. Chitosan has been already used in combination with SLNs to improve interaction and internalization in corneal cell [100], for oral administration of peptides [101] and to decrease SLN internalization by macrophage [102].

**Figure 25:**
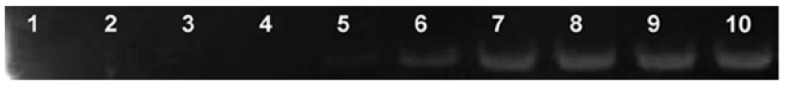
Agarose gel electrophoresis of **17**-based SLNs incubated with increasing concentrations of plasmid DNA (values are expressed in mg mL^−1^) [98].

To confirm the absorption of the first layer of DNA at the surface, the SLNs were incubated with increasing concentrations of plasmid DNA. Electrophoresis (Figure 25) showed that for concentrations up to 5μg/mL, DNA was not detectable. At the higher concentration, the detectable bands correspond to the non-adsorbed DNA.

**Figure 26:**
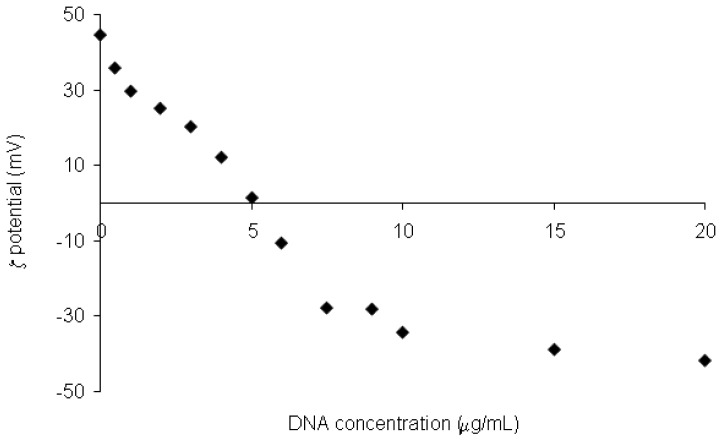
ζ-potential values for **17**-based SLNs incubated with an increasing amount of DNA [98].

Moreover, the study of the electrophoretic mobility (ζ potential) showed that the surface potential values of the coated SLNs with the DNA decreased from +44 to −42 mV when the amount of DNA increased from 0 to 20 μg/mL (Figure 26).

**Figure 27:**
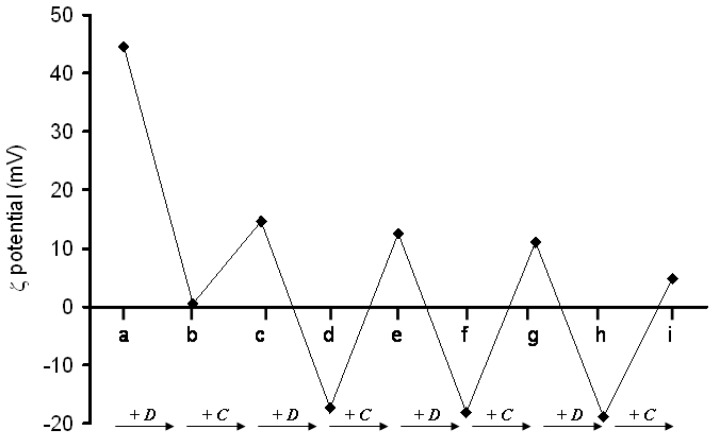
ζ-potential values during the LbL formation; +D: DNA addition; +C: chitosan addition [98].

In addition, it could be seen that at 5 μg/mL of DNA a slight positive value of +1 mV was occurred [98]. LbL coating was studied using ζ-potential measurements; the results (Figure 27) revealed that the value increases from +1 mV after the first layer of DNA to +14 mV after the addition of the chitosan layer. The value successively decreased and increased after consecutive additions of DNA and chitosan.

**Figure 28:**
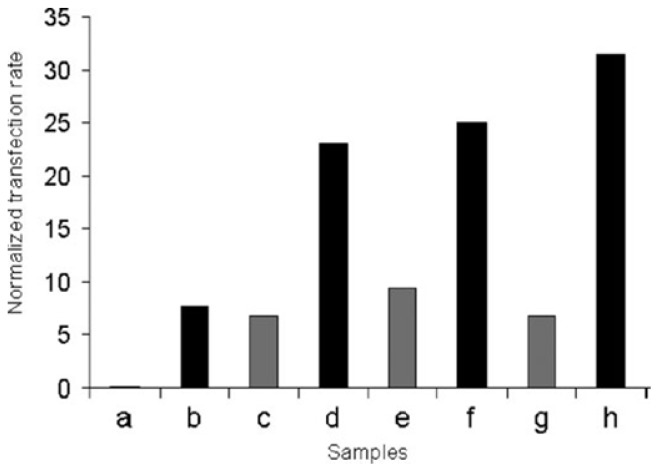
Transfection rates of MDCK cells, normalized with the values obtained for lipofection experiments, using SLNs coated with DNA (**a**); DNA + chitosan: DC (**b**); D–C–D (**c**); D–C–D–C (**d**); D–C–D–C–D (**e**); D–C–D–C–D–C (**f**); D–C–D–C–D–C–D (**g**); and D–C–D–C–D–C–D–C (**h**); black bars represent the SLNs having the chitosan as the last layer [98].

Cell transfection was studied by incubation of coated SLNs with MDCK (Madin-Darby canine kidney Cells). The transfection rates were evaluated by the fluorescence of a green fluorescent protein (GFP) for which the loaded DNA codes and compared to the transfection rates obtained with a reference method (Lipofection). The results presented in Figure 28 showed that higher transfection rates were obtained when the SLNs possess a chitosan layer at their surface (b, d, f and d). In addition, the transfection rate increases when the number of layers increases. Also, there was no relevant change in terms of cell viability, demonstrating a lack of toxicity of these coated SLNs at the studied concentration.

**Figure 29:**
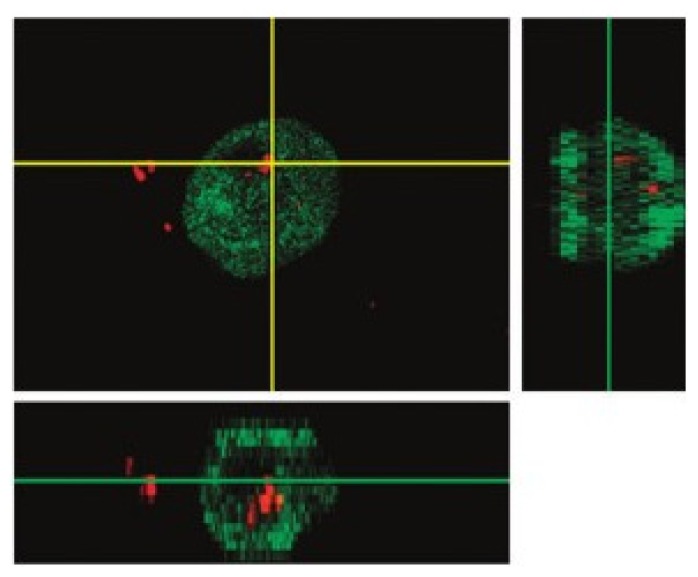
Confocal micrograph of a transfected cell that has expressed green fluorescent protein (GFP). The red color representing the labeled-chitosan appeared to be in the cytoplasmic compartment of the cell [98].

Cell transfection was visualized by coupling chitosan to a fluorescent dye (tetramethylrhodamine) and the cell was then imaged by confocal microscopy. The results presented in Figure 29 show that the SLNs represented by the red color are present in the cytoplasm of the transfected cell close to the nucleus.

#### 2.4.3. SLNs of *Para*-Carboxy-Substituted-Calix[4]arenes, **18**

**Figure 30:**
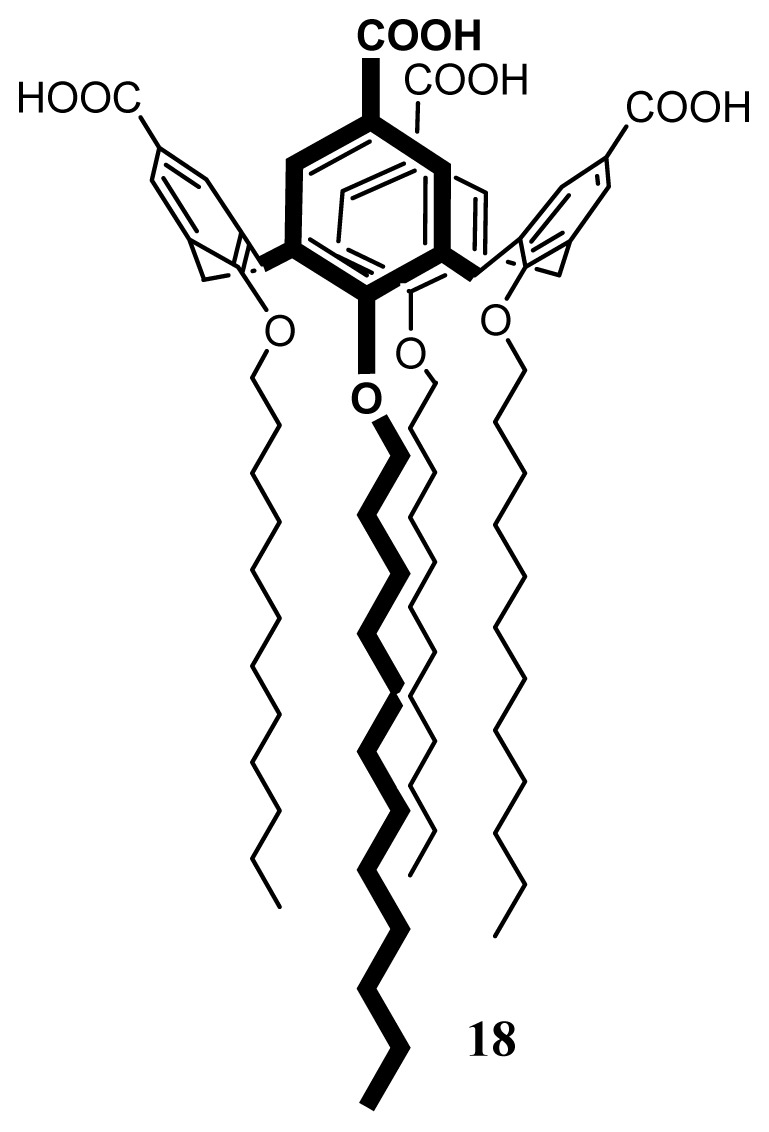
Chemical formula of 5,11,17,23-tetracarboxy-25,26,27,28-tetradodecyloxycalix[4]arene.

Amphiphilic 5,11,17,23-tetra-carboxy-25,26,27,28-tetradodecyloxycalix[4]arene (**1**) (Figure 30) was synthesized from the corresponding tetra-bromo compound as described previously [103].

**Figure 31:**
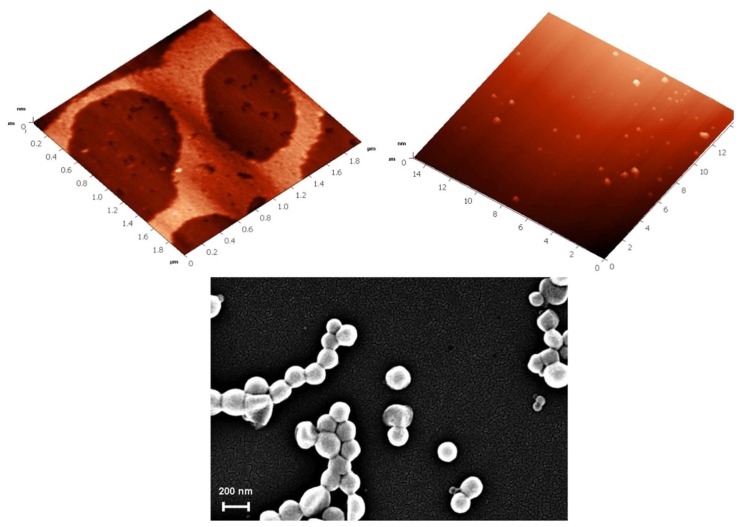
AFM images of a suspension of **18** spread on a mica surface (**top**) at scan ranges of 15 (**left**) and 2 mm (**right**) and SEM images of **18**-based SLNs [104].

Formulation of SLNs based on **18** was carried out using the solvent displacement method [44,48]. The suspension obtained was then characterized by PCS, AFM and Scanning Electron Microscopy (SEM) [104]. The results of AFM and SEM are presented in Figure 31. The AFM images show that the particles co-existed in the form of two different types. The first ones were round objects having a diameter around 200 nm and heterogeneous in size, with only moderate flattening similar to the SLNs based on other amphiphilic calixarene derivatives [45], but different to vesicular structures based on the same amphiphilic compound [105]. The second type of structure showed a height of 1.5–2 nm, which looked very similar to layered structures [104,106]. From the SEM image, it can be clearly observed that the particles have a solid structure resistant to the high vacuum used for imaging.

#### 2.4.4. SLNs of an Apparently Non-Polar-Calix[4]arenes Derivative, **19**

**Figure 32:**
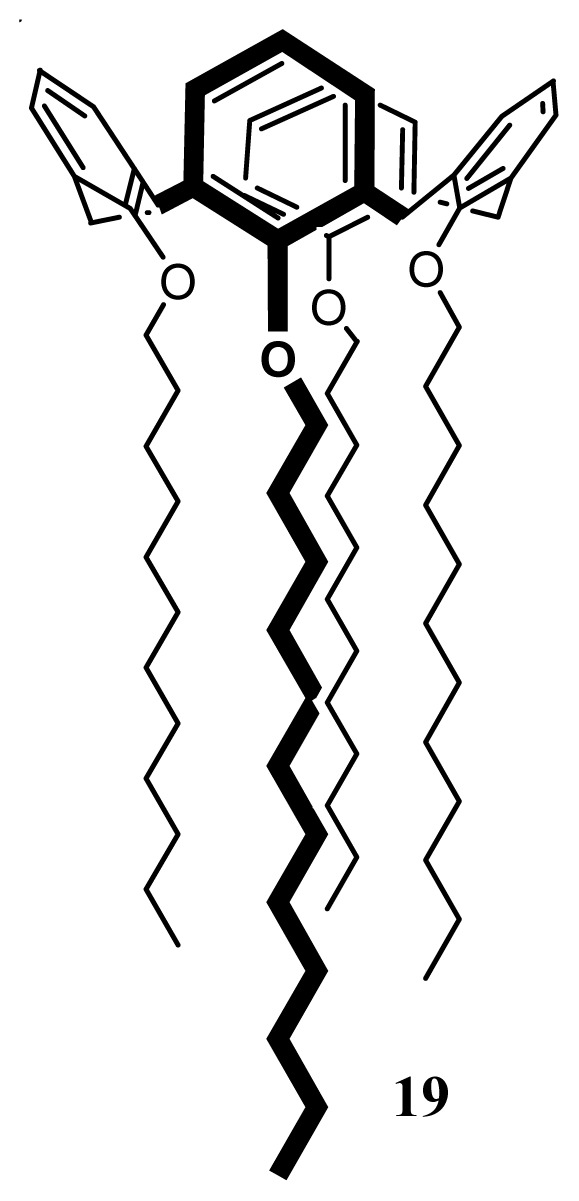
Molecular formula of para-*H*-tetra-*O*-dodecyl-calix[4]arene **19**.

*Para*-*H*-tetra-*O*-dodecylcalix[4]arene (**19**) (Figure 32) was synthesized from the native calix[4]arene; **1** by reaction of sodium hydride and bromododecane in dimethylformamide [107].

**Figure 33:**
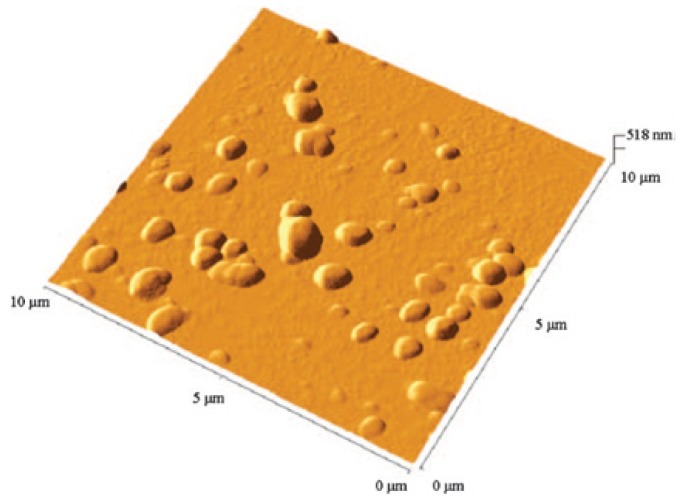
Non-contact mode AFM image of **19**-based nanoparticles ([107]).

SLNs were prepared using the solvent displacement method [44,48] and were characterized by PCS and AFM imaging [107]. The results showed a hydrodynamic diameter of 235 nm and stability over 45 days. Non-contact AFM imaging (Figure 33) showed particles of diameter 400 nm and a height of 130 nm.

Since this type of colloidal particles are based on a calix[4]arene derivative without polar groups, the nano-structure organization of this compound was assumed to be due to interaction between the macrocycle and water [106], this auto-organisation was related to the fact that this structure is able to form hydrogen bonds between the aromatic head groups and the hydroxyl groups of water (π….H–O) as well as non-permeable hydrophobic layer due its alkyl chains [107].

## 3. Solid Lipid Nanoparticles of Calix-Resorcinarenes

### 3.1. SLNs of Resorcinol-Dodecanal Cyclotetramer, **20**

**Figure 34:**
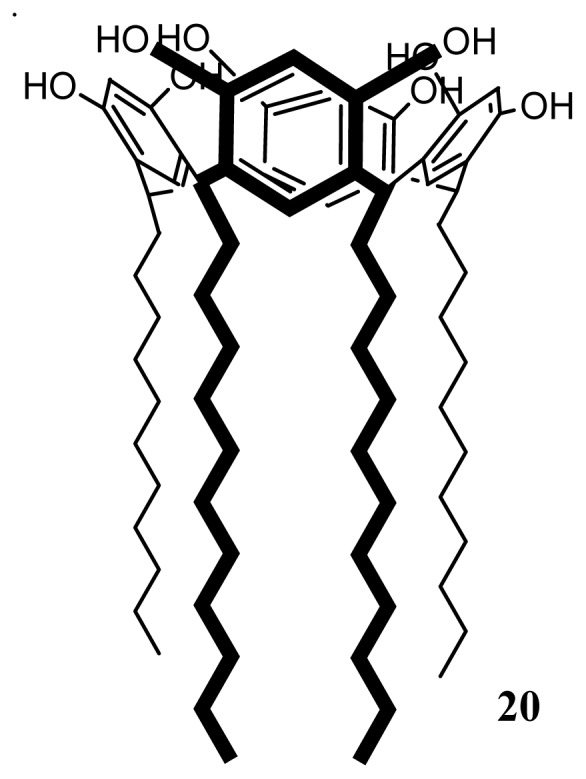
Formula of *c-2*, *c*-8, *c*-14, *c*-20-tetraundecylclix[4]resorcinarene **20**.

Resorcinol-dodecanal cyclotetramer, **20** (Figure 34) was synthesized in a one step procedure as previously described using the acid-catalyzed reaction of resorcinol with dodecanal in ethanol [108].

**Figure 35:**
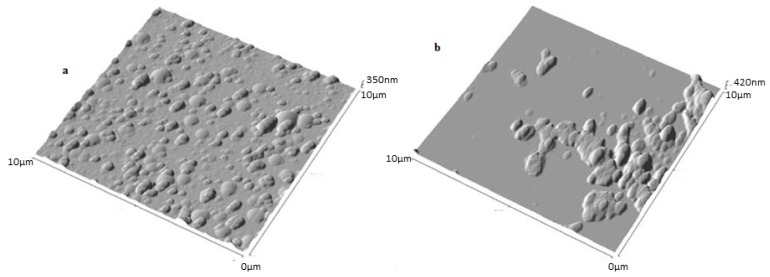
Non-contact mode AFM images of **20**-based SLNs on glass (**a**) and on mica (**b**) at 10 μm scan ranges [109].

SLNs based on **20** were prepared using the solvent displacement method [44,48]. The suspension was characterized by PCS and AFM imaging [109]. PCS gave a hydrodynamic diameter of 150 nm with high monodispersity. Non-contact mode AFM imaging revealed: (i) when the suspension was deposited on a glass substrate (Figure 35a), the presence of round objects of 236 ± 40 nm in diameter and 145 ± 40 in height with a slightly flattened aspect as per the *para*-acylcalix[4]arenes **2a**–**d**[45]. This is in contrast to β-cyclodextrin derivatives that collapse under such condition [110], here when the suspension was deposited on mica substrate the colloidal system appeared as flattened ovoid objects with two population of 210 ± 20 nm and 350 ± 50 nm in diameter, and 90 ± 20 nm in height (Figure 35b).

**Figure 36:**
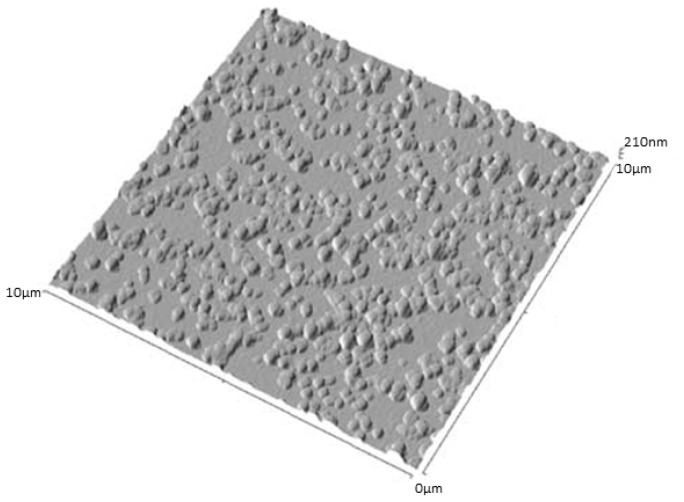
Non-contact mode AFM images of SLNs based on mixtures of **20** and pluronic^®^ F68 acid (10%) at 10 × 10 μm scan range [109].

From the above result, an SLN suspension was formulated with 10% (*w/w*) of surfactant (Pluronic^®^ F68) in the organic phase. The AFM image on a glass substrate showed less particle aggregation with 280 ± 20 nm in diameter and 80 ± 10 nm in height (Figure 36). This result was ascribed to the fact that the surfactant is adsorbed at the surface of the particles and prevents aggregation via steric stabilization [111].

In order to optimize the formation of this kind of SLNs, different studies were undertaken [109]. Since these SLNs can be obtained without surfactant and contrast to other colloidal systems where a surfactant is necessary in their formulation [112], it was of interest to study the possible effects of added surfactant. The result revealed that at 2% (*w/w*) of Pluronic^®^F68 in the organic phase, a small but significant increase in the particle size was observed giving a diameter of 180 nm. Above this amount, the size remained invariant when the surfactant is increased up to 20% (*w/w*). Generally, high concentration of surfactant reduces the surface tension and facilitates the colloidal particle partition by reducing in particle size that is connected to an increase in the surface area [113]. Here, the key difference in the behavior of surfactant toward these SLNs systems could be related to the influence of the hydrophilic-lipophilic balance (HLB) of each amphiphilic supramolecular structure [109] that affects their self-emulsifying proprieties [114].

Regarding the influence of the viscosity of the aqueous phase, varying concentrations of glycerol were used. The results show that at low concentrations of glycerol, the size increases and this peaks at 4% size decreasing to the value found in the case of pure water (150 nm) at 10% of glycerol. This complex result is in contrast to the observations in the cases of **2d**[48] and the **9a**[79] where the size is independent on the viscosity of the aqueous phase. As in the of case **2d** and **9a**, SLNs based on **20** remain stable in term of particle sizes in the pH range of 4 to 8; however lower stability was observed at pH between 2 and 4. The stirring speed had no significant effect on the particle size. This result was in contrast to the observation recently evoked in the case of polymeric nanoparticles [111].

At final concentrations of SLNs above 100 mg/L the diameter increased progressively to reach a value of 180 nm at final concentration equal to 300 mg/L. This result was in contrast to other calix[n]arene derivatives, where the size increases up to 300 mg/L in the case of **2d**[48] and up to 500 mg/L in the case of **9a**[79]. Regarding polymeric nanoparticles produced by the solvent displacement process, it has been proposed that the viscosity of the organic phase was highly dependent on polymer concentration even at lowest value [115], and the particle size increases when the polymer concentration increases [111]. In addition, aggregation occurred at the maximum concentration used [116].

The situation in the case of calix[n]arenes and calix[4]resocinarenes derivatives is drastically different, and could be ascribed to the fact that the particle size control is under two different mechanisms: (i) at low concentration (*i.e.*, below the critical concentration), the size increases when the concentration of amphiphilic increases (ii) above the critical concentration, the particles are dispersed with the same mass and the particle size remains invariant.

For future applications, the effects of various sodium and potassium salts at 0.1 M on the suspension stability were studied [109]. The results revealed that the particle size remains stable in the case of potassium carbonate, potassium sulphate and sodium acetate; however, a slight but significant increase of particle size was observed in the case of sodium carbonate, sodium sulphate and potassium acetate. This result was in contrast to the observations in the cases of **2d**[48] and the **9a**[79] where no significant change in terms of particle size was found except with potassium chloride at 150 mM for **9a**[79]. Precipitation of the suspension occurred immediately in the case of sodium and potassium chloride.

The effect of the microwave irradiation, ultrasonic and thermal treatment was also tested and showed no change in the stability [109]. This observation was in agreement with the result found in the case of the SLNs based on **2a**–**d** and **9a**–**e**[79]; therefore, SLNs based on **20** can be sterilized for intravenous administration and introduced in topical or cosmetic formulation where thermal treatment is needed.

Freeze-drying of SLNs based on **20**[109] was carried out as described above in the case of SLNs based on **2d**[56]. Unexpectedly, the re-dispersion of the lyophilized SLNs did not lead to a complete formation of the native colloidal suspension even at the highest sugar concentration tested (25%, *w/w*); additionally the observed polydispersity was very high. This result was ascribed to the fact that **20** possesses a high affinity for the carbohydrates [117,118] and did not allow the reconstitution of the SLNs or may favor the restructuration of the colloidal particles into aggregated structures. This phenomena, was related to the presence of multiple hydroxyl groups with fixed nature arrangement in the structure that increase the efficient sugar affinities by hydrogen bonding [117].

### 3.2. SLNs of Modified Calix-Resorcinarenes

#### 3.2.1. SLNs of Tetrakis(*N*-methylprolyl)tetraundecylcalix[4]resorcinarenes (l-RA-Pro), **21**

**Figure 37:**
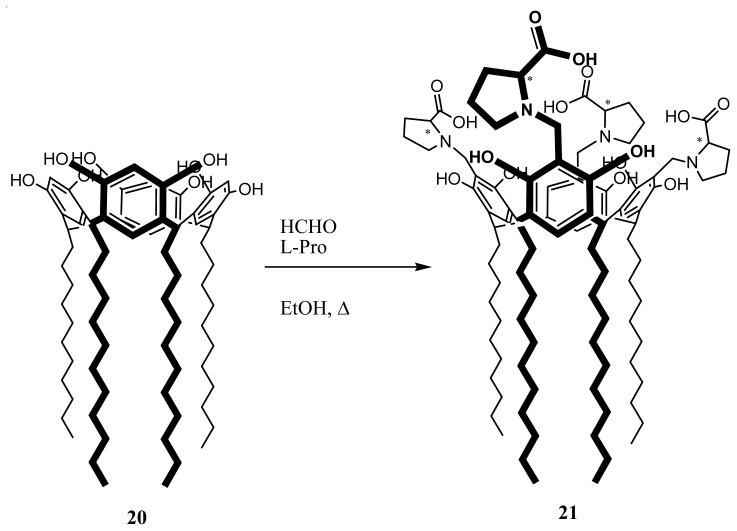
Synthetic route to l-RA-Pro **21**.

Amphiphilic l-RA-Pro, **21** were synthesized via a Mannich reaction of tetraundecylcalix[4]resorcinarene, **20**[108], l-proline and formaldehyde in ethanol (Figure 37) [119].

**Figure 38:**
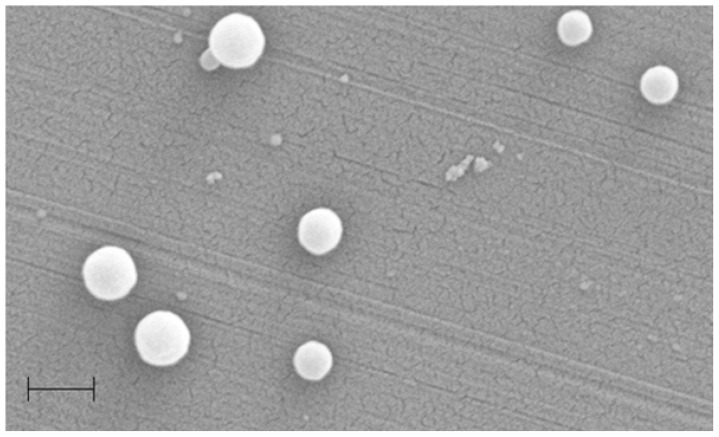
Scanning electron miroscopy image of l-RA-Pro-based SLNs spread on a glass surface (scale bar 200 nm) [120].

**Figure 39:**
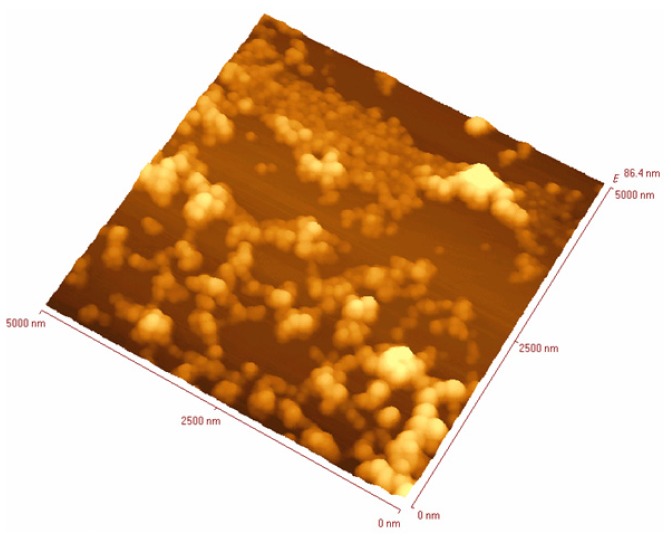
Non-contact mode AFM image of l-RA-Pro-based SLNs spread on a freshly cleaned glass slide [120].

SLNs based on **21** were formulated via the solvent displacement method [44,48] and characterized using PCS [120], showing a diameter of 195 ± 5 nm. Under the same condition, SLNs based on parent supramolecular structure, **20** were prepared with a particle size of 200 ± 5 nm. The SLNs were also characterized using SEM and non-contact mode AFM imaging (Figures 38 and 39).

**Figure 40:**
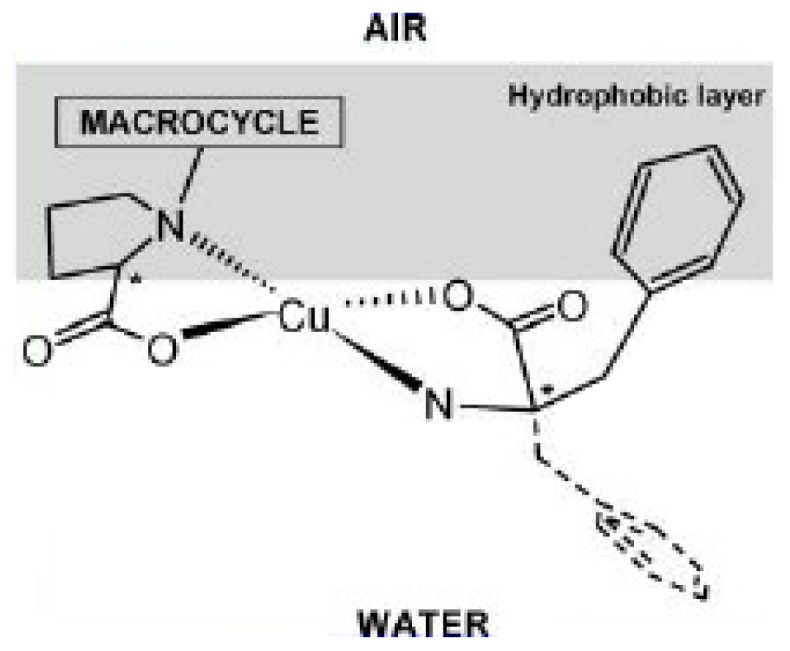
Schematic representation of the proposed interaction mechanism. The lateral chain of Phe is represented by a dashed line for the l-enantiomer (For clarity, only the prolyl moiety of l-RA-Pro is represented) [121].

The amphiphilic properties of **21** have been investigated previously [121] the molecule forms stable Langmuir monolayers at the air-water interface. It was also demonstrated that the prolyl moiety is immerged in water and able to interact with copper (II) and l-phenylalanine (l-Phe) to form enantioselective complexes (Figure 40). The authors assumed that this propriety was conserved in the case of the SLNs and that the prolyl moiety was accessible at their surface.

**Figure 41:**
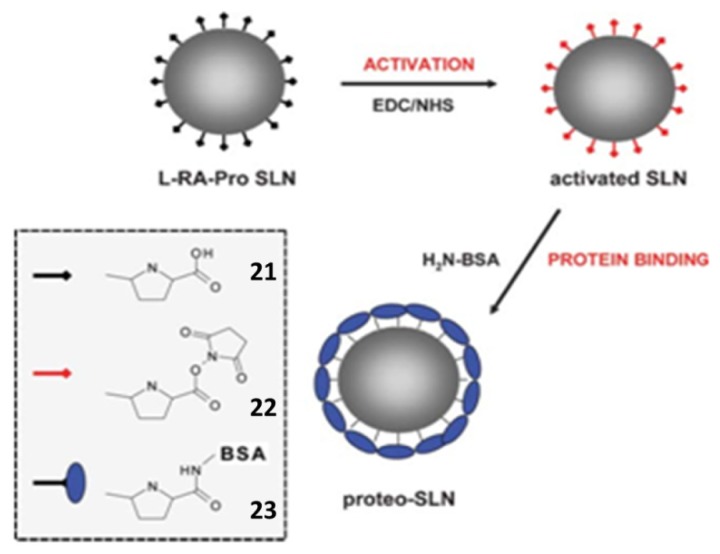
Synthetic route to proteo-SLNs **23**[120].

SLNs based on **21** were chemically activated using hydroxysuccimide, **22** and then reacted with bovine serum albumin (BSA) to yield proteo-SLNs [120], **23** (Figure 41).

**Figure 42:**
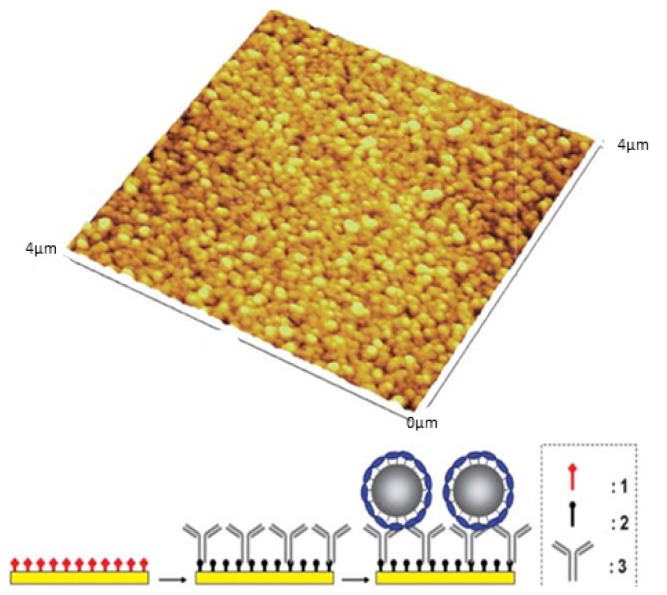
Non-contact mode AFM of proteo-SLNs immobilized on a gold surface via antibody-antigen interactions and schematic representation of the strategy used to attach the SLNs on the surface (**1**: activated ester; **2**: deactivated amide functions, **3**: antibody anti-BSA) [120].

PCS analyses of these particles showed no significant change in term of particle size after chemical modification and the value was 200 ± 5 nm. Furthermore, non-contact mode AFM imaging was undertaken to confirm this result. In this case, anti-BSA antibodies were immobilized on functionalized gold, and then incubated with proteo-SLNs suspension [120]. The observed results (Figure 42) reveal that the SLNs form a uniform monolayer on the surface, with absence of aggregation already observed with **21** based SLNs (Figure 39). This observation was attributed to the specific interaction between the proteo-SLNs and anti-BSA antibodies immobilized on the gold surface. The particles were slightly flattened on the surface with a diameter of 210 ± 20 nm and height of 170 ± 20 nm; these results were in good agreement with the PCS results, and show a high degree of rigidity for these SLNs.

**Figure 43:**
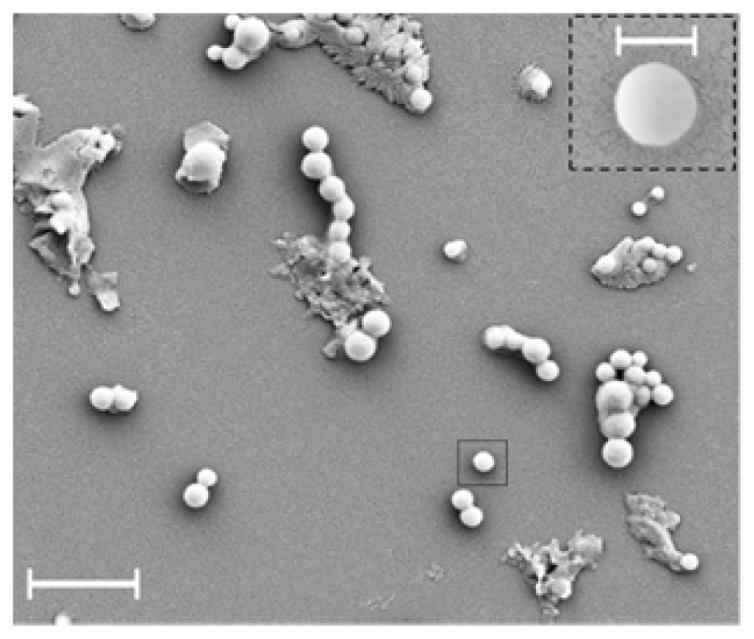
Scanning electron microscopy image of proteo-SLNs spread on a glass substrate (scale bars 1 μm and 200 nm (inset)) [120].

SEM imaging has also been used and the result is presented in Figure 43. It could be seen that the proteo-SLNs are localized at the surface as round objects and measurement reveals a diameter 220 ± 34 nm, and are in perfect agreement with the results obtained with AFM and PCS experiments, confirming the rigid solid structure.

#### 3.2.2. SLNs of Resocinarene Bis-Crown Ethers (CNBC5), **24**

**Figure 44:**
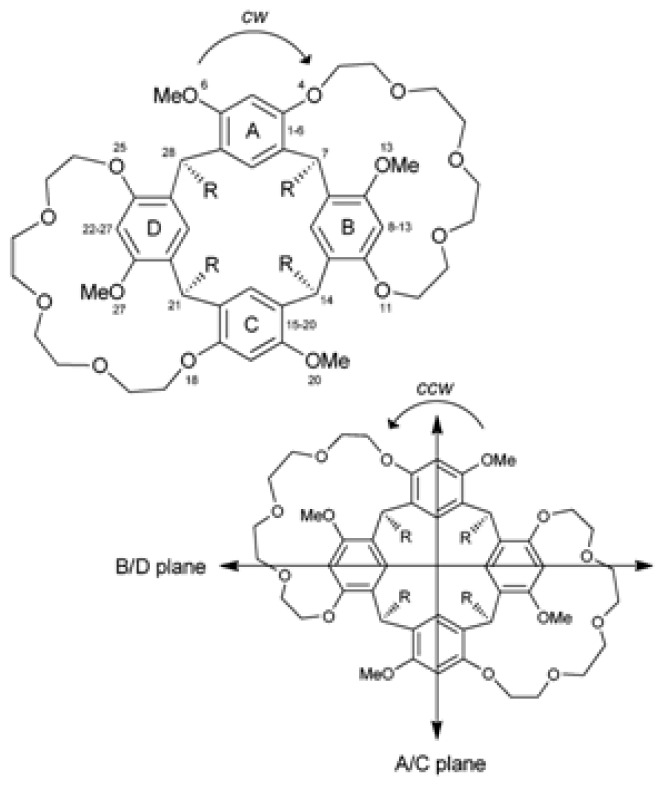
Structure of the resorcinarene bis-crowns CNBC5 **24**, R = C*_n_*H_2_*_n_*_+1_ where *n* = 2, 3, 4, 5, 7, 9, 10, 11 with selected crystallographic numbering. cw and ccw enantiomers are shown; the A/C plane runs through the upright aryl rings (A and C) and the B/D plane through parallel aryl rings (B and D) respective to the methine plane C7–C14–C21–C28 [124].

CNBC5, **24**, (Figure 44) where N represents the number of carbons in the alkyl groups (R) at the lower rim, and 5 is the number of oxygen atoms in each polyether bridge, has been synthesized by reaction of a suitable ethylene glycol with various tetramethoxy resorcinarenes [122], prepared using Lewis acid catalyzed condensation of the 3-methoxyphenol with different aldehydes [123].

Tetramethoxy resorcinarene bis-crown ethers (C3BC5, C4BC5, C5BC5, C7BC5 C9BC5, C10BC5 and C11BC5) syntheses were carried out using a cesium carbonate mediated coupling with a suitable tosylated oligo-ethylene glycol [124].

**Figure 45:**
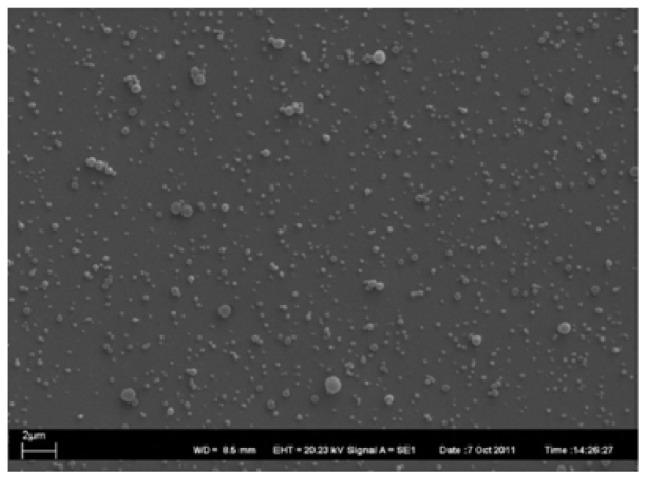
SEM image of C11BC5 SLN on SiO*_x_*; spherical particles with a mean diameter of 300 nm are shown [124].

SLNs suspension based on **C11**–**24** was prepared using the solvent displacement method [44,48] in the absence of surfactant. The suspension was then characterized by PCS and SEM [124]. The obtained results showed a hydrodynamic diameter of 220–320 nm and the SEM image revealed spherical particles with a size distribution corresponding to the DLS measurements (Figure 45).

**Figure 46:**
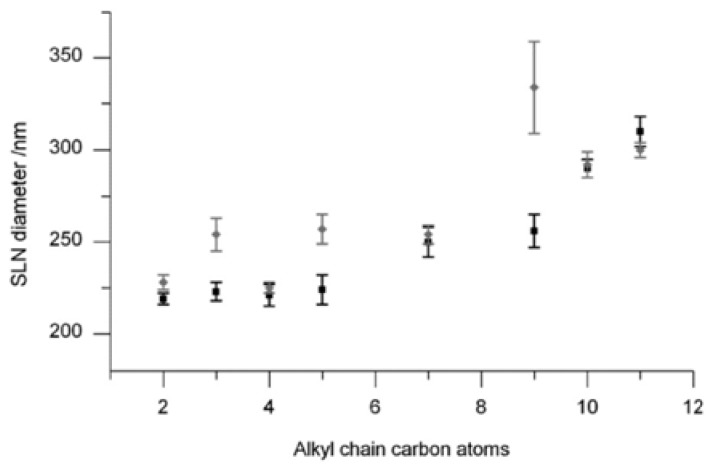
SLN diameters for all CNBC5 **24** (mean of the size distribution from DLS showing standard deviation). SLNs with a constant 0.1 mM concentration (**black**) and with a constant mass 100 mg L^−1^ (**grey**) show increasing diameter for longer alkyl chains [124].

The effect of length of the alkyl chain and the final concentration on the particle sizes of the suspension has been studied [124]. The result represented in Figure 46 showed that in the first series with a constant molar concentration of **24**, the particle sizes increases when the length of alkyl chain increases. In the second series the concentration was kept constant in mg/L, the particle size was similar than the first one. This observation indicated that the change in the amount of the CNBC5 between the two series did not affect the proprieties of the SLNs suspensions, and the variation of the particle size depended only on the length of alkyl chain.

This result was in contrast to the observation obtained in the case of **9a**–**e**[79] where the size decreases with the acyl chain length and in the case of **2a**–**d**[48] where the particle size remains invariant when the acyl chain length increases. The authors explained these results by the fact that calix[9]arene is a larger macrocyclic ring, with more conformation flexibility compared to calix[4]arene and calix[4]resorcinarene, which can lead to different arrangement of the amphiphilic during its self-assembly.

## 4. Conclusions

After the first study of the SLNs based on *para*-acylcalix[4]arenes, several other supramolecular structures based on calix[n]arenes and calix-resorcinarenes have been investigated to generate other SLNs. All these colloidal nano-structures showed a great similarity to the SLNs based on commercial lipids in term of stability and thus show potential for future applications. Despite the suspected toxicity of the phenolic compounds and the current lack of FDA (Food and Drug Administration) approval to use these amphiphilic macrocycle molecules in medicine, the encouraging preliminary results given in this review in terms of *in-vitro* toxicity and stability in biological fluids allow us to expect that these types of colloidal nanostructures deserve further preclinical and clinical medical investigations.

## Figures and Tables

**Table 1:** Lipids commonly used in the preparation of solid lipid nanoparticles.

Lipid	Formula	Melting point
Triacylglycerols (Ester derive from glycerol and three fatty acids)	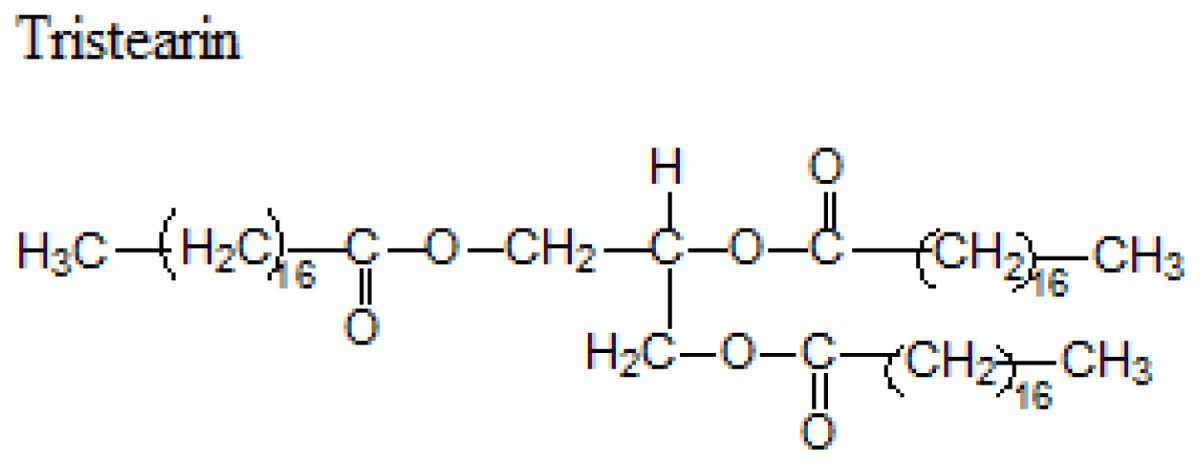	55 °C
	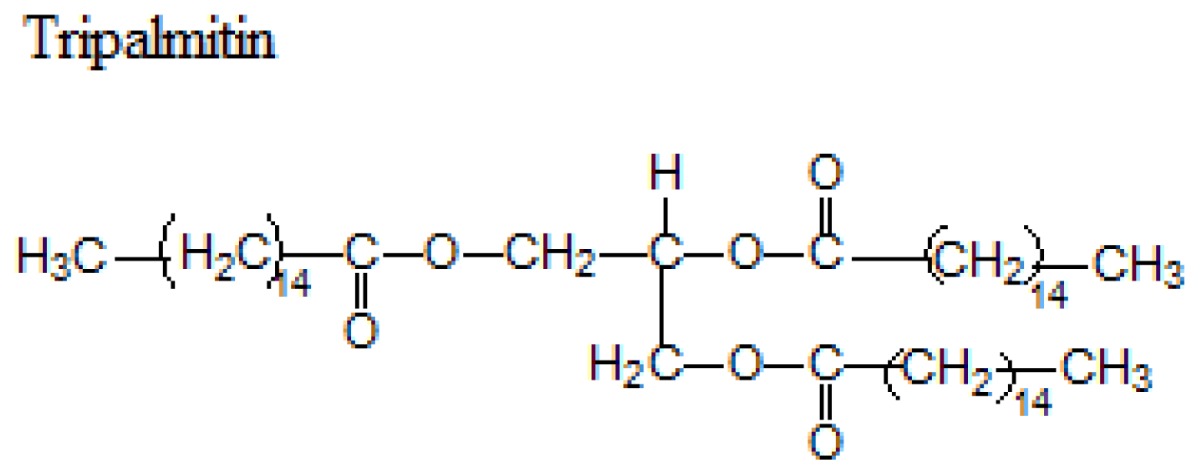	66–68 °C
	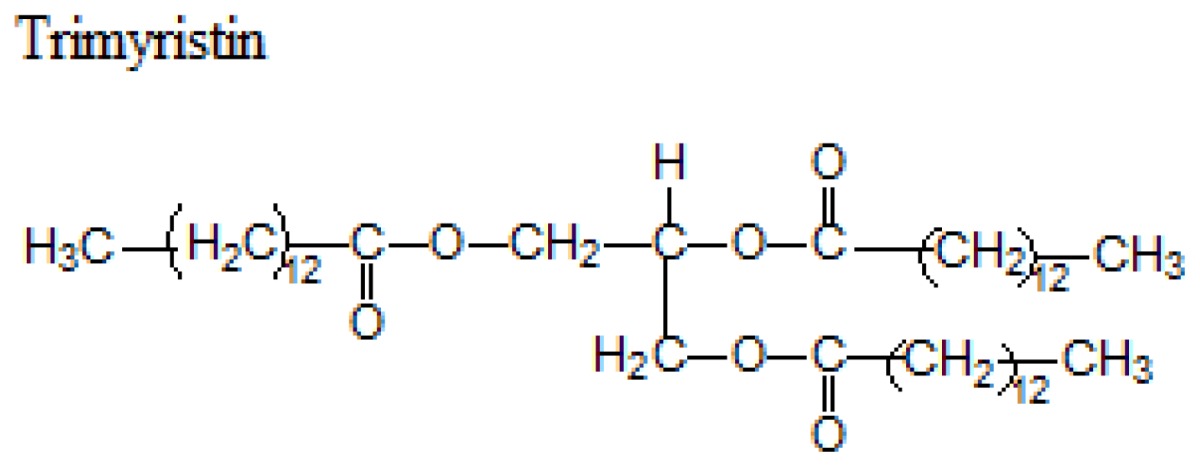	56–57 °C

Acylglycerol mixtures (Ester of the alcohol glycerol in which a hydroxyl group is replaced by a fatty acid)	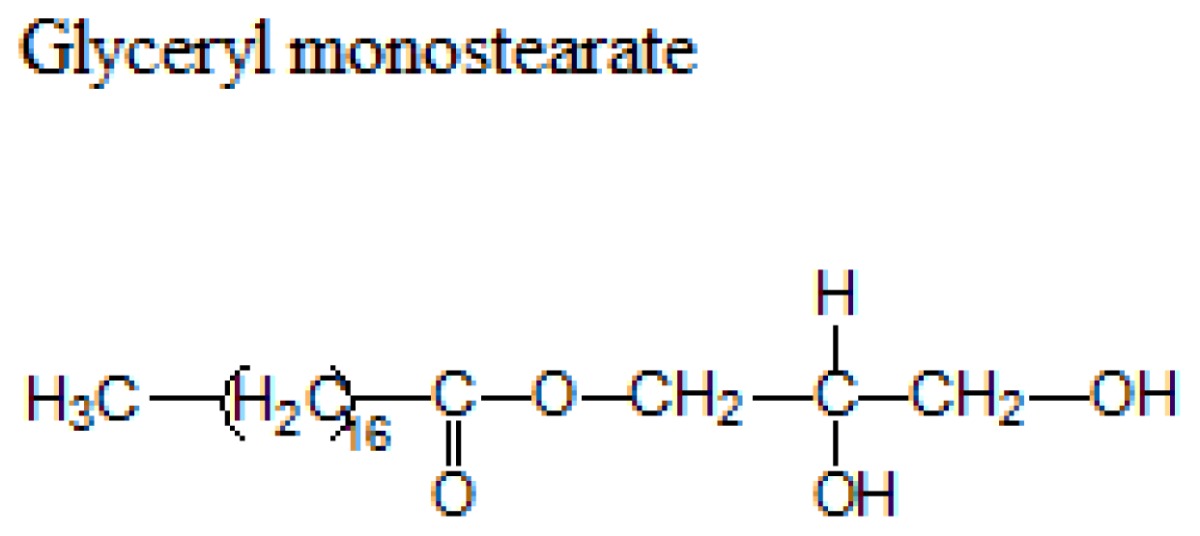	58–59 °C
	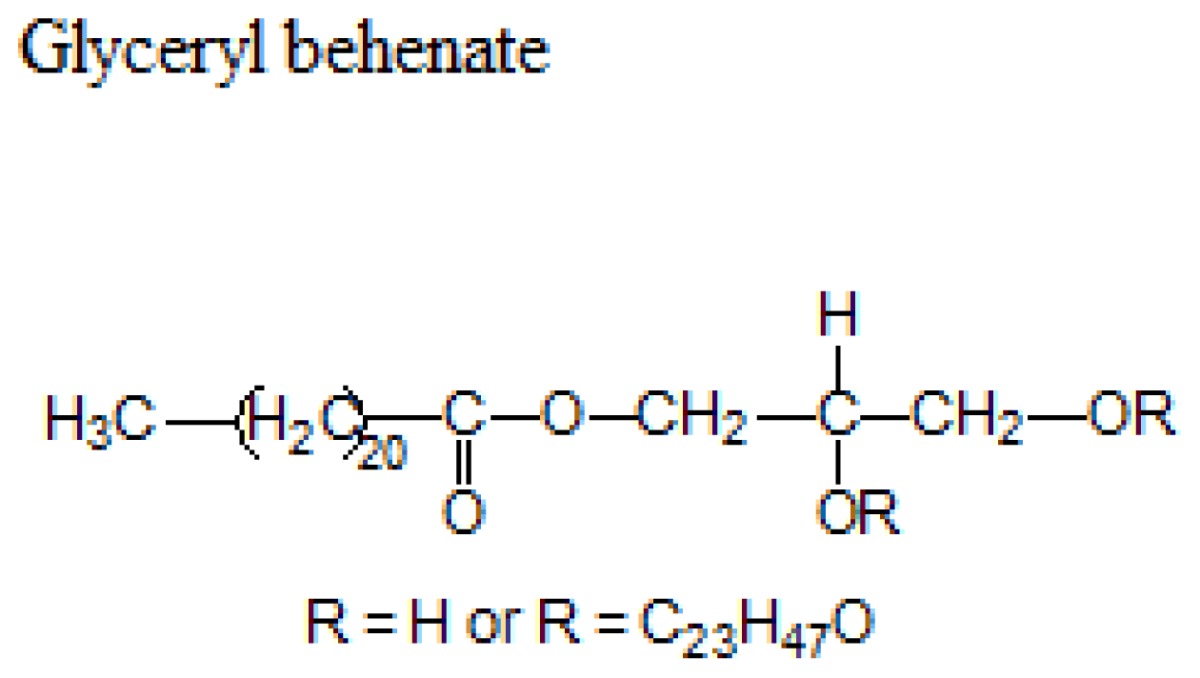	83 °C
	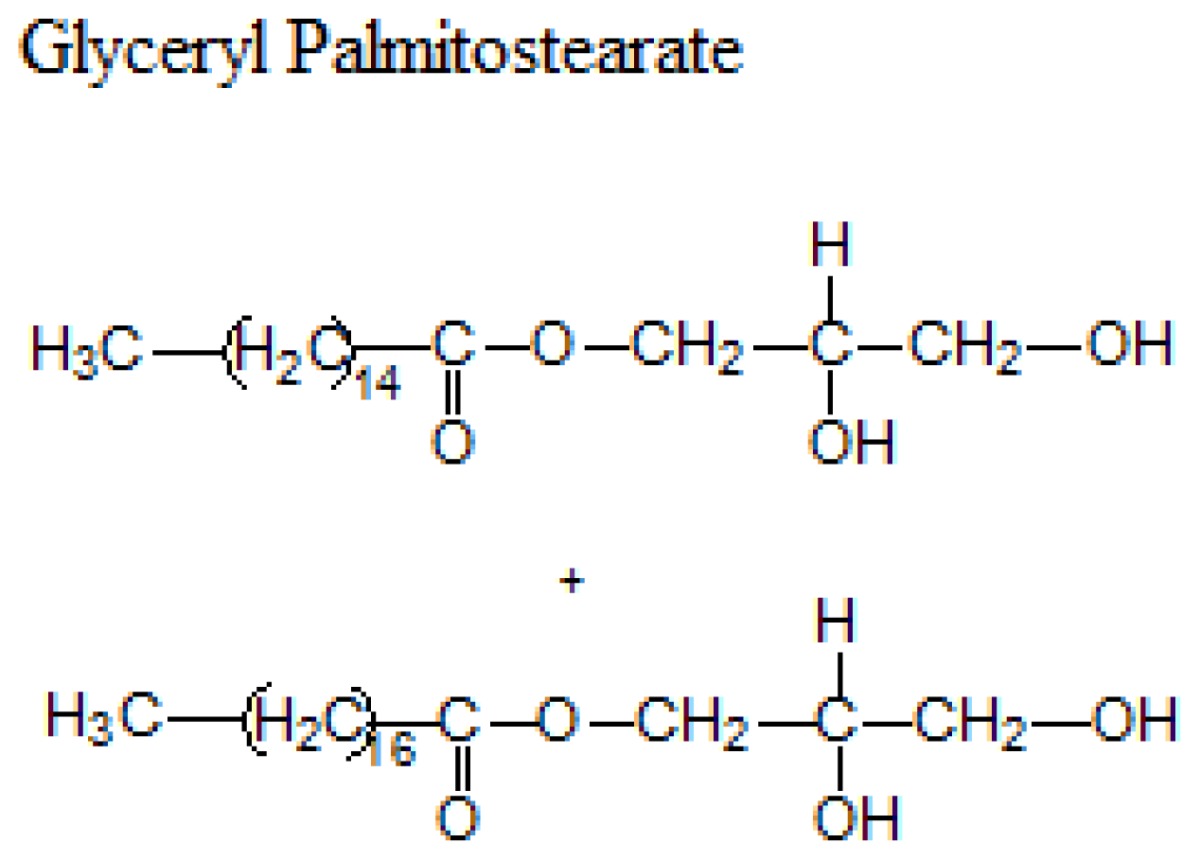	56 °C

Waxes (Ester of a long chain alcohol and a fatty acid)	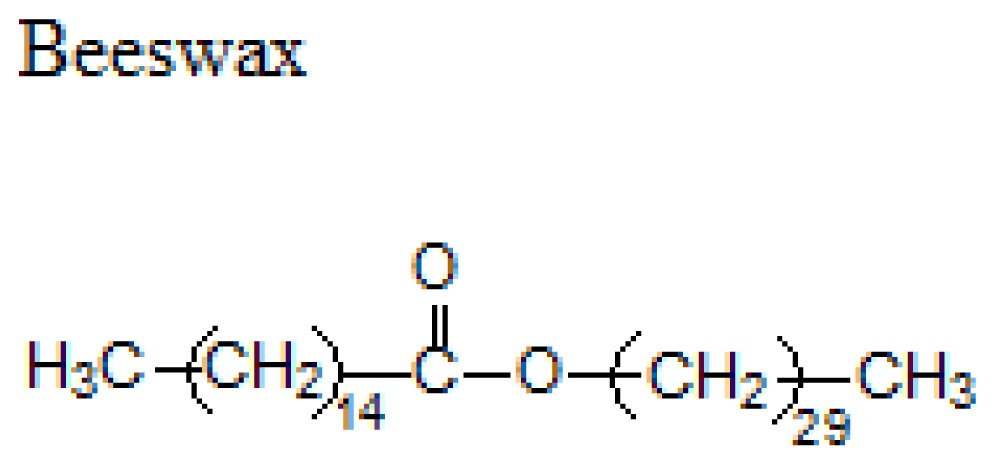	62–64 °C
	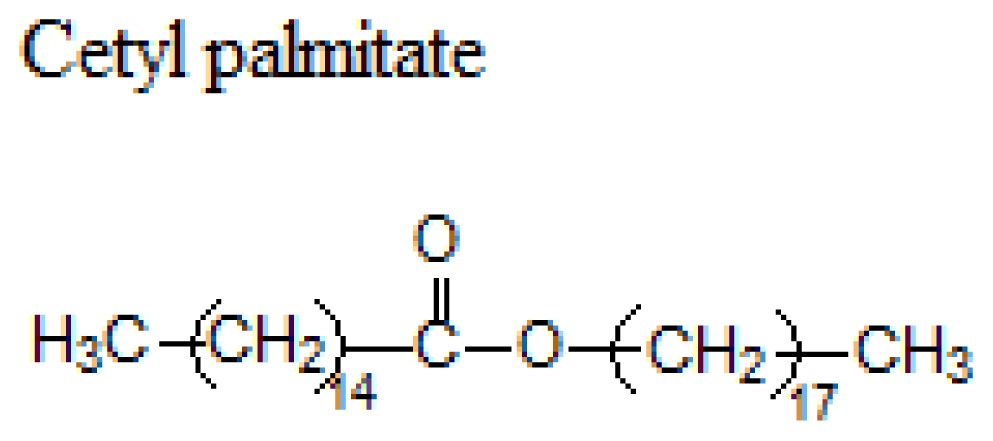	54 °C

Hard Fats (Saturated fatty acid)	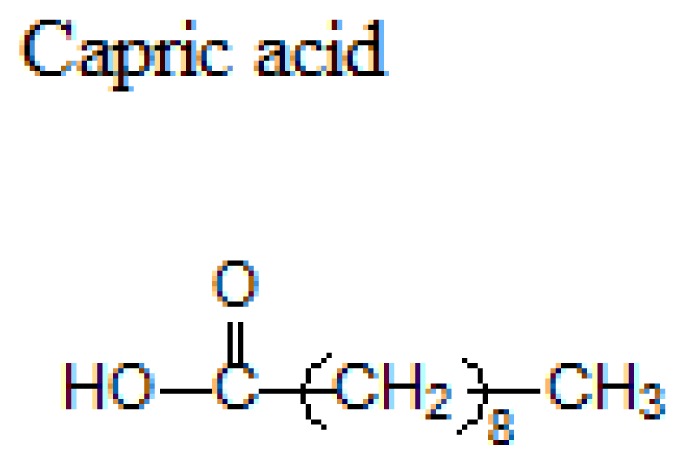	32 °C
	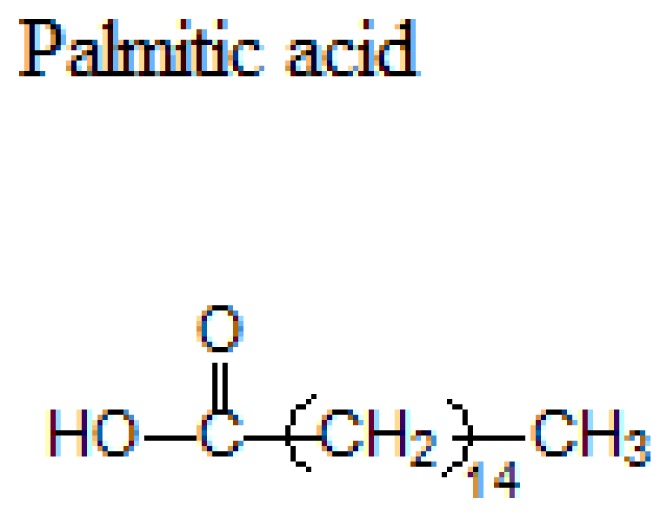	63 °C
	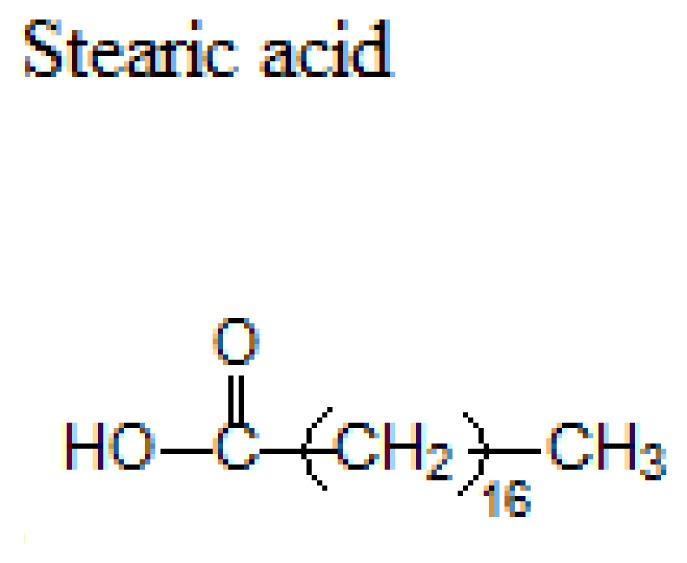	70 °C
	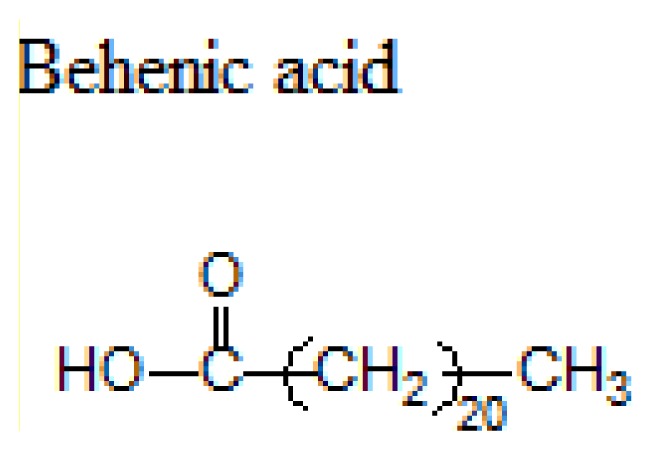	80 °C

**Table 2:** Hydrodynamic diameter of the different *para*-acylcalix[4]arenes based SLN_(s)_ prepared in phosphate buffer saline (PBS) measured by photon correlation spectroscopy (PCS).

Compound	2a	2b	2c	2d	3d	4d
Average hydrodynamic diameter (nm)	340	353	350	350	325	260

**Table 3:** Results of the haemolysis experiments (expressed as a percentage of total haemolysis caused by hypotonic stress) at calix-arene SLN concentrations varying from 50 to 150 mg/L, values are derived from the O.D. measured at 540 nm.

Concentration mg/L of SLNs	2a	2b	2c	2d	3d	4d
50	0.0	0.0	0.5	0.0	0.0	1.8
75	0.0	3.2	0.0	0.0	0.0	0.0
100	0.0	0.5	0.0	0.0	0.25	0.0
125	0.0	0.0	0.0	0.0	0.62	0.0
150	0.0	0.0	0.0	0.0	0.0	0.0
